# Symmetry breaking unlocks superior reactivity in single atom catalytic therapy

**DOI:** 10.1016/j.mtbio.2025.102464

**Published:** 2025-10-27

**Authors:** Chenyu Ding, Chengzhong Du, Penghui Wei, Yongrui Hu, Hongjia Zheng, Zhongyuan Shen, Peisen Yao, Lingjun Yan, Yang Zhu

**Affiliations:** a Department of Neurosurgery, Neurosurgery Research Institute, The First Affiliated Hospital, Fujian Medical University, Fuzhou, Fujian, 350005, China; b The First Affiliated Hospital, Binhai Campus of the First Affiliated Hospital, Fujian Medical University, China

**Keywords:** Single-atom enzyme, Ferroptosis, Catalytic therapy

## Abstract

Single atom enzymes (SAEs) serve as promising alternatives to natural enzymes in therapeutic applications due to their simple structure and strong catalytic performance. However, their activity often lags native enzymes, limiting their practical utility. Although synergistic strategies involving auxiliary drugs or molecular partners can improve therapeutic outcomes, the added complexity hinders translational feasibility. Therefore, enhancing SAE reactivity without introducing additional components remains a critical challenge. Here, inspired by the asymmetric coordination architectures of natural metalloenzymes, we introduce a biomimetic Mn-S_1_N_3_ coordination environment to break the intrinsic symmetry of conventional Mn-N_4_ SAEs. The incorporation of a single sulfur atom triggers localized electronic polarization, reconfigures the Mn orbital alignment, and generates an internal electric field that facilitates intermediate adsorption and bond activation. This coordination tuning significantly lowers the energy barrier for the oxidation of hydrogen peroxide and glutathione, leading to enhanced catalytic performance. Both experimental and DFT studies demonstrate that Mn-S_1_N_3_ outperforms its symmetric Mn-N_4_ counterpart, effectively driving irreversible tumor ferroptosis. This work presents a symmetry-breaking design strategy that bridges the performance gap between artificial and natural enzymes, providing a streamlined and translatable solution for single atom catalytic therapy.

## Graphical abstract

Herein, we successfully fabricate asymmetric Mn-S_1_N_3_/SAE with isolated Mn-S_1_N_3_ active sites and a symmetric Mn-N_4_/SAE with isolated Mn-N_4_ active sites. The low electronegativity heteroatom of sulfur optimizes the adsorption and desorption of oxygen-involving intermediates. As anticipated, asymmetric Mn-S_1_N_3_/SAE exhibits superior peroxidase- and glutathione oxidase-like activities compared to symmetric Mn-N_4_/SAE, highlighting the role of S coordination in lowering the energy barrier.

**Figure:**
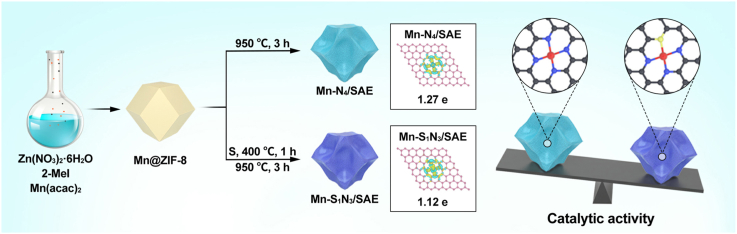

## 1 Introduction

Single atom nanozymes (SAEs) have attracted growing attention as potential stand-alone alternatives to natural enzymes in biomedicine [[1], [2], [3], [4], [5]]. Their atomically dispersed metal centres not only offer well-defined catalytic sites and full metal utilization, but also address several limitations of natural enzymes, including poor stability, high production cost and limited shelf life [[6], [7], [8], [9], [10], [11], [12]]. These advantages make SAEs particularly suitable for therapeutic applications [11,[13], [14], [15], [16], [17], [18]]. Among these, nitrogen (N)-doped carbon frameworks anchoring single metal atoms with symmetric metal-N_4_ coordination have been extensively studied due to their well-defined structures and synthetic feasibility [[19], [20], [21], [22], [23], [24]]. However, their catalytic activity remains inferior to that of natural metalloenzymes, primarily because the symmetric coordination environment limits electron localization and weakens interactions with key oxygen-containing intermediates such as O∗, OH∗, and OOH∗ [[25], [26], [27]]. Reducing the coordination number, such as the construction of metal-N_3_ sites, can improve reactivity, but often results in insufficient binding with hydrogen peroxide, causing premature intermediate release and diminished catalytic efficiency [[28], [29], [30], [31], [32]]. While synergistic strategies that combine SAEs with external agents or therapeutic partners can enhance performance, such approaches add formulation complexity and hinder clinical translation [[33], [34], [35]]. Therefore, enhancing the intrinsic catalytic activity of SAEs while maintaining structural simplicity remains a central challenge.

Inspired by the asymmetric coordination architectures found in natural metalloenzymes [[36], [37], [38]], heteroatom modulation may be a powerful approach to enhance the single atom catalysis. Introducing atoms such as sulfur (S) [39,40], boron (B) [[41], [42], [43]], or phosphorus (P) [7,44] into the coordination environment has been shown to lower energy barriers in key reaction steps. In particular, S coordination enhances charge polarization at the active site, strengthens interactions with reactants like hydrogen peroxide (H_2_O_2_), while also stabilizing the active site by slightly adjusting metal-ligand bond lengths [19,45,46]. These modifications help mitigate the premature desorption of intermediates, enhance charge transfer, and increase overall catalytic performance.

Building on this concept, we engineered Mn-based SAEs with either symmetric Mn-N_4_ or asymmetric Mn-S_1_N_3_ coordination. The larger atomic radius of S caused distortion from the ideal coordination plane in Mn-S_1_N_3_, further tuning the adsorption strength of reactive intermediates. As a result, Mn-S_1_N_3_ displayed enhanced peroxidase- and glutathione oxidase-like activities, effectively converting H_2_O_2_ into hydroxyl radicals (‧OH) and oxidizing GSH to GSSG. Density functional theory calculations reveal that the asymmetric Mn-S_1_N_3_ configuration increases electron localization, strengthens adsorption of H_2_O_2_ and glutathione (GSH), and lower the activation barrier for catalysis. These effects are further supported by shortened Mn-N bond lengths under S coordination, which facilitates more effective charge transfer. Both *in vitro* and in vivo experiments demonstrated that Mn-S_1_N_3_ effectively induces irreversible tumor ferroptosis through lipid peroxidation (LPO) and glutathione peroxidase 4 (GPX4) inactivation. The catalytic performance was further enhanced under 808 nm laser irradiation, leading to marked inhibition of tumor growth. These findings establish coordination asymmetry as a key design principle for improving the catalytic efficiency of SAEs and promoting their application in future catalytic therapy.

## 2 Results

**Fig. 1:**
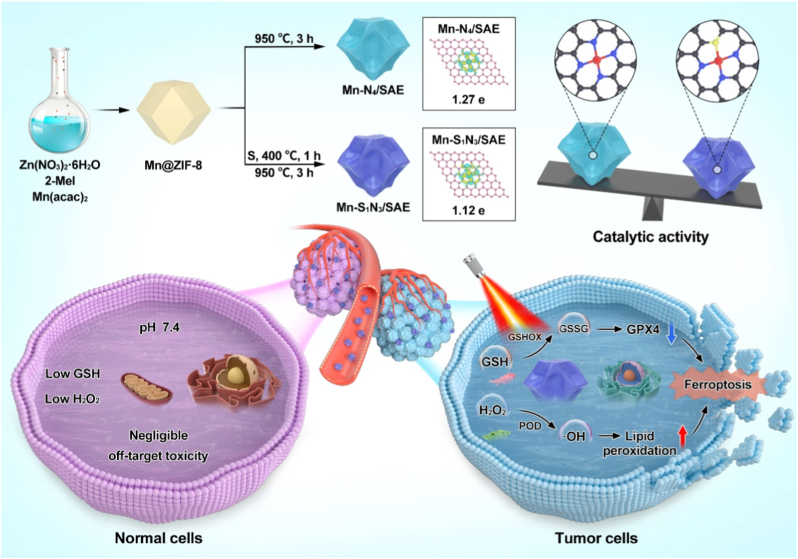
Schematic diagram illustrating the synthesis process of Mn-based SAEs, s-doping-induced asymmetric coordination, and catalytic-triggered tumor ferroptosis.

**Fig. 2:**
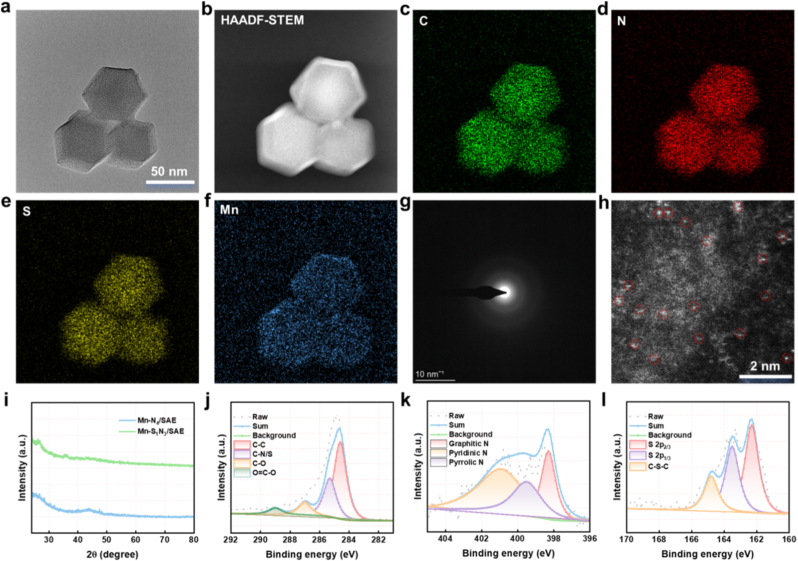
**Characterization of Mn-S_1_N_3_/SAE.** (a) TME image of Mn-S_1_N_3_/SAE. (b–f) EDX mapping image of Mn-S_1_N_3_/SAE. (g) SAED image of Mn-S_1_N_3_/SAE. (h) HAADF-STEM image of Mn-S_1_N_3_/SAE. (i) XRD pattern of Mn-S_1_N_3_/SAE. (j) High-resolution C 1s, and (k) N 1s, and (l) S 2p XPS spectra in Mn-S_1_N_3_/SAE.

The synthetic route of Mn-based SAEs was demonstrated in Fig. 1. These Mn-based SAEs were synthesized via a controlled carbonization process starting with the Mn@ZIF-8precursors. Initially, the Mn@ZIF-8 precursors were synthesized using a host-guest strategy, where manganese acetylacetonate (Mn(acac)_2_) was encapsulated in situ with the ZIF-8 host cage. The Mn@ZIF-8 derivatives retained a rhombic dodecahedron morphology (Fig. S1). Upon simply annealing the Mn@ZIF-8 precursor, we successfully obtained an Mn-N_4_/SAE (Fig. S2). In contrast, Mn@ZIF-8 precursor was mixed with sublimed S and pyrolyzed at 400 °C for 1 h, followed by 950 °C for 3 h in an argon atmosphere to fabricate Mn-S_1_N_3_/SAE. Transmission electron microscopy (TEM) images revealed that Mn-S_1_N_3_/SAE exhibited a uniform dodecahedron shape, with an approximate diameter of 50 nm (Fig. 2a). The high-resolution TEM further showed that the surface of Mn-S_1_N_3_/SAE became rough and porous following the high-temperature carbonization (Fig. S3). In addition, Raman analysis demonstrated that the ZIF-8 derived N-C framework was poorly crystallized after pyrolysis, with a significant number of defects, which favorably supports the immobilizing of isolated Mn atoms (Fig. S4). The energy-dispersive X-ray spectroscopy (EDX) mapping showed a homogeneous distribution of Mn, C, N, and S atoms within the Mn-S_1_N_3_/SAE (Fig. 2b–f and S5). Selected area electron diffraction (SAED) images of both Mn-S_1_N_3_/SAE and Mn-N_4_/SAE displayed two diffuse diffraction rings, indicative of amorphous N-C frameworks (Fig. 2g and S6). Aberration-corrected atomic-resolution high-angle annular dark-field scanning transmission electron microscopy (HAADF-STEM) directly showed bright dots, highlighted by red circles, representing atomically distributed Mn (Fig. 2h). X-ray diffraction (XRD) pattern demonstrated no crystalline peaks for metallic Mn or Mn oxide nanoparticles, suggesting no significant Mn aggregation in Mn-S_1_N_3_/SAE, supporting the successful creation of atomically dispersed Mn nanozyme (Fig. 2i). The Mn loading efficiency in Mn-S_1_N_3_/SAE and Mn-N_4_/SAE was determined to be 0.68 % and 0.72 %, respectively, as measured by inductively coupled plasma mass spectrometer (ICP-MS). These results proved successful construction of both asymmetric and symmetric Mn-based SAEs. Next, we further evaluated their surface electrical properties and stability in biological media. As shown in Fig. S7, the zeta potentials of Mn-S_1_N_3_/SAE and Mn-N_4_/SAE in water showed negative charge. After dispersing the Mn-S_1_N_3_/SAE in DMEM buffer (pH 7.4), fetal bovine serum, and whole blood, and incubating at 37 °C for different time periods (0, 12, 24, 48 h), the particle size changes were minimal (Fig. S8). These results indicate that both Mn-S_1_N_3_/SAE possess excellent stability under physiological conditions, providing a safety guarantee for their potential biomedical applications.

**Fig. 3:**
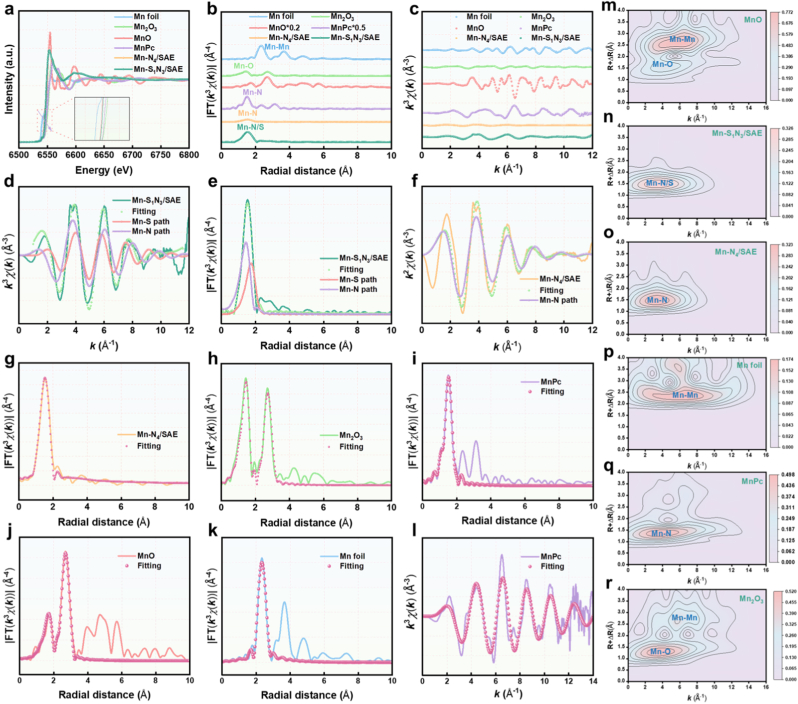
**Atomic structural analysis of Mn-N_4_/SAE and Mn-S_1_N_3_/SAE.** (a) Mn K-edge XANES spectra of Mn foil, MnPc, Mn_2_O_3_, MnO, Mn-S_1_N_3_/SAE, and Mn-N_4_/SAE (inset: the enlarged pre-edge region). (b) The Fourier transform EXAFS of the Mn K-edge of Mn foil, MnPc, Mn_2_O_3_, MnO, Mn-S_1_N_3_/SAE, and Mn-N_4_/SAE. (c) EXAFS curves of Mn foil, MnPc, Mn_2_O_3_, MnO, Mn-S_1_N_3_/SAE, and Mn-N_4_/SAE at the k space. (d) EXAFS fitting curves of Mn-S_1_N_3_/SAE, (f) Mn-N_4_/SAE, and (l) MnPc at the k space. (e) EXAFS fitting curve of Mn-S_1_N_3_/SAE, (g) Mn-N_4_/SAE (h) Mn_2_O_3_, (i) MnPc, (j) MnO, and (k) Mn foil at the R space. (m) Wavelet transformation of Mn K-edge EXAFS of MnO, (n) Mn-S_1_N_3_/SAE, (o) Mn-N_4_/SAE, (p) Mn foil, (q) MnPc, and (r) Mn_2_O_3_.

To investigate the binding states of Mn, S, and N atoms in Mn-S_1_N_3_/SAE and Mn-N_4_/SAE, X-ray photoelectron spectroscopy (XPS) measurements were performed (Figs. S9 and S10). As shown in Fig. 2j and S11, the high-resolution C 1s spectrum of the Mn-based SAEs predominantly exhibited peaks corresponding to sp^2^-hybridized graphitic C, associated with C-C, C-N/S, and C-O bonds. The high-resolution N 1s spectrum revealed three distinct N species: graphitic N, pyridinic N, and pyrrolic N. Notably, a high content of pyridinic N species was observed, which provided coordination sites for immobilizing single-atomic Mn, potentially strengthening the catalytic activity of Mn-based SAEs (Fig. 2k and S12). The high-resolution S 2p spectrum in Mn-S_1_N_3_/SAE exhibited well-defined peaks at S 2p_3/2_, S 2p_1/2_, along with a peak corresponding to C-S-C, slightly contracting the Mn-N bond length (Fig. 2l). In addition, Mn 2p_3/2_ peak at 640.8 eV was positioned between the Mn^0^ (637.9 eV) and Mn^3+^ (642.3 eV) peaks, indicating the atomic Mn^δ+^ nature (0 <δ < 3) state in the Mn-based SAEs (Fig. S13). To further explore the coordination environment of Mn, synchrotron radiation-based X-ray absorption near-edge structure (XANES) and extended X-ray absorption fine structure (EXAFS) analyses were conducted. The Mn K-edge XANES spectra of both Mn-S_1_N_3_/SAE and Mn-N_4_/SAE demonstrated an energy absorption threshold between that of Mn foil and Mn_2_O_3_ (Fig. 3a), suggesting the Mn^δ+^ (0 <δ < 3) oxidation state, consistent with the XPS results. Furthermore, the Fourier transform of the Mn-N_4_/SAE EXAFS data in R-space showed a clear peak at 1.50 Å, corresponding to the Mn-N bond. In contrast, Mn-S_1_N_3_/SAE EXAFS data revealed a peak at 1.56 Å, corresponding to both Mn-N and Mn-S bonds. Furthermore, no significant peak was observed about 2.66 Å, which proved Mn - Mn bond, further indicating the isolated Mn active sites in Mn-based SAEs (Fig. 3b and c). EXAFS fitting analysis at the Mn K-edge offered well-defined structural information, demonstrating that the Mn atoms in Mn-N_4_/SAE were coordinated with four N atoms, while the Mn atoms in Mn-S_1_N_3_/SAE were coordinated with one S atom and three N atoms (Fig. 3d–l, S14, and Table S1). In addition, wavelet transform (WT) analysis was applied to further confirm the presence of atomically dispersed Mn species. As expected, both Mn-S_1_N_3_/SAE and Mn-N_4_/SAE showed WT signals at 3.4 Å^−1^ and 3.51 Å^−1^, respectively, corresponding to the Mn-N or Mn-S bond, with no WT intensity observed for Mn - Mn bonds (Fig. 3m–r). These results conclusively demonstrated the successful fabrication of Mn-based SAE with atomically dispersed Mn-N_4_ or Mn-S_1_N_3_ active sites.

**Fig. 4:**
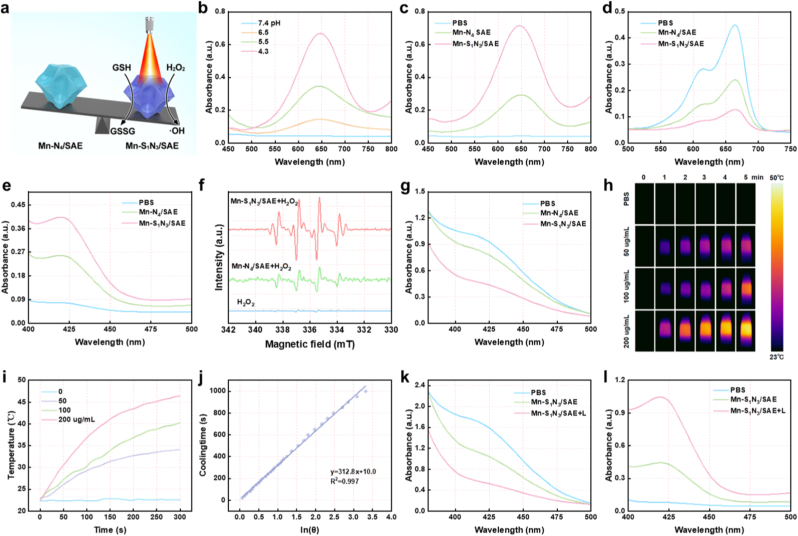
**Enzymatic performance of Mn-N_4_/SAE and Mn-S_1_N_3_/SAE.** (a) Schematic representation of the catalytic properties of Mn-S_1_N_3_/SAE. (b) UV-vis spectra of TMB incubated with Mn-S_1_N_3_/SAE and H_2_O_2_ at various pH. (c) UV-vis spectra of TMB incubated with H_2_O_2_ in the presence of varying formulations. (d) The UV-vis spectra of MB treated with varying formulations in the presence of H_2_O_2_. (e) The UV-vis spectra of ABTS treated with varying formulations in the presence of H_2_O_2_. (f) The ESR curves of •OH captured using a DMPO. (g) UV-vis spectra of DTNB incubated with varying formulations in the presence of GSH. (h) The infrared thermal (IT) imaging of various different concentrations of Mn-S_1_N_3_/SAE. (i) The photothermal effect of Mn-S_1_N_3_/SAE upon 808 nm laser irradiation. (j) The cooling curve of Mn-S_1_N_3_/SAE. (k) GSH elimination capacity of Mn-S_1_N_3_/SAE under 808 nm laser irradiation. (i) Evaluate the catalytic activity of Mn-S_1_N_3_/SAE under laser irradiation by ABTS assay.

Inspired by the presence of polyvalent Mn atoms, we investigated the catalytic performances of Mn-based SAEs (Fig. 4a). The POD-mimicking activity of Mn-based SAE was evaluated by monitoring the absorbance at 652 nm using 3,3’-5,5’-tetramethylbenzidine (TMB) as a probe for •OH generation [47]. As shown in Fig. 4b, Mn-S_1_N_3_/SAE induced a rapid alteration in TMB absorbance within the acidic pH range of 4.0–6.0, while exhibiting no significant alteration at a neutral pH of 7.4 (Fig. S15). This suggested that Mn-S_1_N_3_/SAE catalyze the reaction in a concentration-dependent manner (Figs. S16 and S17). Notably, the absorbance change in TMB was significantly faster for Mn-S_1_N_3_/SAE than that of Mn-N_4_/SAE at an acidic pH value of 4.3, indicating a higher catalytic activity of Mn-S_1_N_3_/SAE (Fig. 4c). In addition, we conducted a TMB assay comparing Mn-S_1_N_3_/SAE with other reported nanozymes (Cu_2_O, Fe_3_O_4_, Mn_3_O_4_). As shown in Fig. S18, Mn-S_1_N_3_/SAE exhibited significantly higher POD-like activity, indicating its superior catalytic efficiency. Furthermore, the kinetics of the as-prepared SAEs, the Km and Kcat were measured. The Mn-S_1_N_3_/SAE showed higher Kcat and lower Km compared to Mn-N_4_/SAE, demonstrating the superior catalytic activity (Fig. S19). To further assess the POD-like activity, the degradation of methylene blue (MB) was monitored. As depicted in Fig. 4d and S20, Mn-S_1_N_3_/SAE effectively facilitated a significant decrease in absorbance at 662 nm in the presence of H_2_O_2_, indicating efficient catalysis of H_2_O_2_ to •OH under acidic conditions and superior enzymatic activity compared to Mn-N_4_/SAE. 2,2’-azino-bis(3-ethylbenzothiazoline-6-sulfonic acid) diammonium salt (ABTS) was employed to further prove the generation of •OH. As shown in Figs. S21 and S22, Mn-S_1_N_3_/SAE triggered a higher enhancement in absorbance compared to Mn-N_4_/SAE when ABTS was treated with H_2_O_2_, further suggesting that S doping amplified catalytic performance (Fig. 4e). To monitor this reaction, we employed 5,5-dimethyl-1-pyrrole oxide (DMPO) as a spin trap for •OH radicals. Exposure to H_2_O_2_ resulted in an electron spin resonance (ESR) spectrum exhibiting a distinctive quartet signal corresponding to the DMPO/•OH intermediate (1:2:2:1) (Fig. 4f). Importantly, Mn-S_1_N_3_/SAE exhibited higher POD-like activity than Mn-N_4_/SAE, underscoring the influence of S doping on the catalytic performance of the central metal atom. In addition, the GSH depletion capacity of Mn-based SAEs was investigated using 5,5′-dithiobis-(2-nitrobenzoic acid) (DTNB) kit (Fig. 4g–S23 and S24). The significant reduction in absorbance at 412 nm confirmed the remarkable GSH depletion capacity of both Mn-S_1_N_3_/SAE and Mn-N_4_/SAE, attributing to the excellent catalytic activity of nature Mn^δ+^. As expected, Mn-S_1_N_3_/SAE exhibited a higher GSH depletion ability compared to Mn-N_4_/SAE, highlighting the enhanced catalytic efficacy from S doping. In addition, Mn-S_1_N_3_/SAE also exhibited oxidase-like activity, which convert O_2_ into superoxide anion (•O_2_^−^) (Fig. S25). Beyond catalytic activity, Mn-based SAEs also showed photothermal therapeutic effects. It can be observed from Fig. 4h and i that the temperature of Mn-S_1_N_3_/SAE rapidly increased within 5 min upon 808 nm laser irradiation, while no significant temperature change was observed in the PBS treatment group. Repeated irradiation cycles confirmed that Mn-S_1_N_3_/SAE showed impressive photothermal stability after three cycles (Fig. S26). The photothermal conversion efficiency (PCE) of Mn-S_1_N_3_/SAE was measured to be h = 31.5 % based on Roper's method (Fig. 4j and S27). Finally, the catalytic activities of Mn-S_1_N_3_/SAE under laser irradiation were assessed using DTNB and ABTS assays. As shown in Fig. 4k,l and S28, Mn-S_1_N_3_/SAE significantly enhanced the GSH depletion and ABTS, TMB free radical generation after laser irradiation, suggesting potential therapeutic benefits. These findings indicated that Mn-S_1_N_3_/SAE exhibited superior POD- and GSHOx-like catalytic activities compared to Mn-N_4_/SAE, with their enzymatic activity further enhanced by 808 nm laser irradiation due to the S-doping of the central metal atom.

**Fig. 5:**
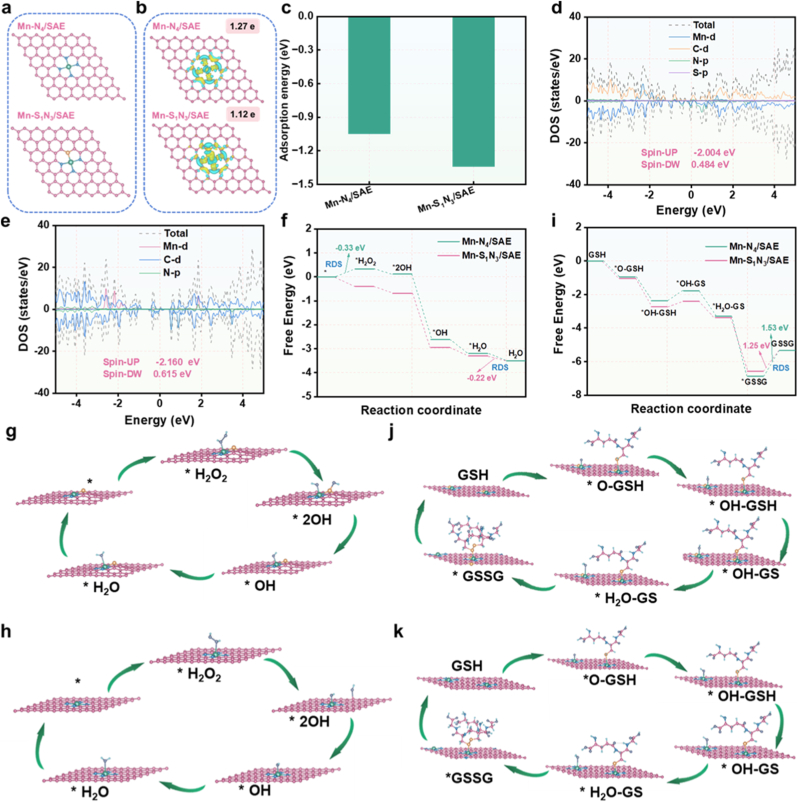
**The DFT calculations of POD- and GSHOx-like activities of Mn-based SAE.** (a) Structures optimization of Mn-S_1_N_3_/SAE and Mn-N_4_/SAE. (b) The charge density difference and Bader charges of Mn-S_1_N_3_/SAE and Mn-N_4_/SAE. (c) Adsorption energies of H_2_O_2_. (d) The DOS of oxygen adsorption state in Mn-S_1_N_3_/SAE and (e) Mn-N_4_/SAE. (f) Gibbs free-energy diagrams for POD- and (i) GSHOx-like activities on modeled surface of both Mn-S_1_N_3_/SAE and Mn-N_4_/SAE. (g) DFT optimized structures of the •OH intermediates on Mn-S_1_N_3_/SAE and (h) Mn-N_4_/SAE surface. (j) DFT optimized structures of the GSSG intermediates on Mn-S_1_N_3_/SAE and (k) Mn-N_4_/SAE surface.

DFT calculations were performed to investigate the geometric and electronic structures of Mn-S_1_N_3_/SAE and Mn-N_4_/SAE. Two structural configurations, namely the symmetric Mn-N_4_/SAE and asymmetric Mn-S_1_N_3_/SAE, were constructed and optimized (Fig. 5a). Analysis of the geometric structure and charge density difference showed that the S coordination caused out-of-plane distortions of the Mn atom and resulted in non-uniform charge distributions. This was attributed to the larger atomic radius and lower electronegativity of S compared to N. Fig. 5b demonstrated a more significant charge accumulation between the doped S and the central Mn atoms, which can be attributed to the electron donation from the S atom. Bader charge analysis proved that the charge on Mn atoms in the asymmetric Mn-S_1_N_3_/SAE (1.12 e) was lower than that in the symmetric Mn-N_4_/SAE (1.27 e), indicating a relatively weaker electron depletion in the former. Furthermore, we calculated the adsorption energies of H_2_O_2_ on both Mn-S_1_N_3_/SAE and Mn-N_4_/SAE models. As anticipated, the Mn-S_1_N_3_/SAE configuration exhibited lower adsorption energy for H_2_O_2_ compared to the Mn-N_4_/SAE configuration, suggesting that S doping amplified the Mn-OH bond, potentially strengthening both adsorption and kinetic reactions with H_2_O_2_. This enhanced adsorption capacity of Mn-S_1_N_3_/SAE was further supported by the shorter Mn-O bond distance. A comparison of the projected electronic densities of states between Mn-S_1_N_3_/SAE and Mn-N_4_/SAE revealed that the d-band center of Mn-S_1_N_3_/SAE shifted closer to the Fermi level compared to Mn-N_4_/SAE, leading to a higher occupation of electrons near the Fermi energy level (Fig. 5c and d). This finding corroborated that the asymmetric Mn-S_1_N_3_/SAE exhibited a stronger Mn-O interaction compared to Mn-N_4_/SAE. Overall, these results confirmed that S doping facilitated the transfer of active electrons and lowered barrier energy for O-involving intermediates during the catalytic process, thereby promoting the production of •OH yield. To evaluate the intrinsic catalytic activities of the symmetric Mn-N_4_/SAE and asymmetric Mn-S_1_N_3_/SAE configurations, Gibbs free energy diagrams were performed for POD- and GSHOx-mimicking activity. For POD-mimicking activity, the optimized H_2_O_2_ readily adsorbed onto the single-atomic Mn (Fig. 5e–g). For Mn-N_4_/SAE, the rate-determining step (RDS) involved the generation of ∗H_2_O_2_, with an energy barrier of −0.33 eV. In contrast, the RDS of Mn-S_1_N_3_/SAE involves the desorption of ∗H_2_O, with an energy barrier of −0.22 eV. This lower energy barrier implied that Mn-S_1_N_3_/SAE exhibited superior POD-like activity than that of Mn-N_4_/SAE. For GSHOx-mimicking activity, optimized GSH spontaneously absorbed onto the single-atomic Mn (Fig. 5h–j). Mechanically, the absorbed GSH∗ and OH∗ reacted to form GS∗ on Mn 3d orbitals, accompanied by H_2_O molecule regeneration. The generated GS∗ then coupled with another GSH molecule to form GSSG. Importantly, the higher energy barrier of GSSG∗ desorption in Mn-N_4_/SAE (1.53 eV) compared with Mn-S_1_N_3_/SAE (1.25 eV) showed that Mn-S_1_N_3_/SAE primarily catalyzed the oxidation of GSH, consistent with the experimental results. These DFT calculations conclusively demonstrated that Mn-S_1_N_3_/SAE outperforms Mn-N_4_/SAE counterparts in both POD- and GSHOx-mimicking activities.

**Fig. 6:**
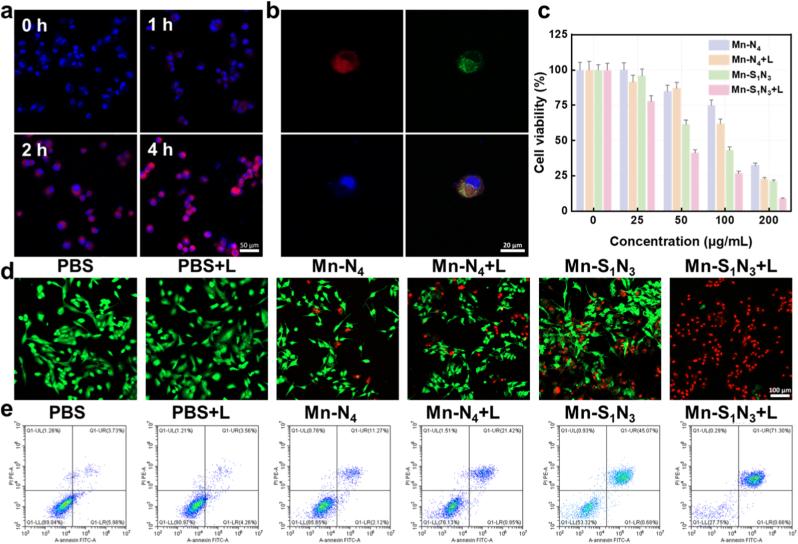
**The therapeutic efficacy on tumor cells.** (a) The CLSM images of U251 cells treated with Cy5.5-labeled Mn-S_1_N_3_/SAE. (b) The CLSM images of U251 cells colocalization. (c) Tumor cell viability after 24 h of incubation with varying concentrations of Mn-S_1_N_3_/SAE combined with laser irradiation. (d) The fluorescence images of live/dead staining for U251 cells incubated with varying formulations. (e) Flow cytometry measurements of U251 cells after incubation with varying formulations.

Due to the combined effect of enzymatic activity and photothermal effect, the cellular cytotoxicity of Mn-based SAEs was assessed using U251 cells. To evaluate cellular uptake, cyanine 5.5 (Cy5.5)-labeled Mn-S_1_N_3_/SAE was utilized, and confocal laser scanning microscopy (CLSM) revealed a time-dependent accumulations in red fluorescence within U251 cells (Fig. 6a and S29). In addition, colocalization study showed that Mn-S_1_N_3_/SAE primarily localized in lysosomes and cytoplasm (Fig. 6b), consistent with the acidic environment required to boost the Mn-S_1_N_3_/SAE-medicated Fenton reaction. The cytotoxic effects of Mn-based SAEs on U251 cells were evaluated using the Cell Counting Kit-8 (CCK-8) kit. Fig. 6c showed the survival rate of U251 cells significantly decreased with increasing concentrations of Mn-based SAEs. As expected, Mn-S_1_N_3_/SAE exhibited a higher inhibitory effect compared to Mn-N_4_/SAE. At a concentration of 200 μg/mL with laser irradiation, the inhibition rate of Mn-S_1_N_3_/SAE against U251 cells reached 91.17 %, significantly higher than the 78.84 % inhibition observed without laser irradiation. Mn-S_1_N_3_/SAE had negligible impact on HT-22, ascribed to significantly low endogenous H_2_O_2_ levels in noncancerous cells (Fig. S30). To further evaluate the antitumor effect of Mn-S_1_N_3_/SAE, a propidium iodide (PI)/calcein-AM co-staining assay was performed. The co-staining results indicated that Mn-S_1_N_3_/SAE produced stronger red fluorescence intensity than Mn-N_4_/SAE, implying a greater degree of cell death (Fig. 6d and S31). And the anti-proliferation effect of Mn-based SAEs was further enhanced upon laser irradiation. Flow cytometry analysis using Annexin V-FITC and PI staining further proved the anti-proliferation effect of Mn-based SAEs (Fig. 6e), showing the highest proportion of cell death in the Mn-S_1_N_3_/SAE plus laser group compared to other groups. These findings collectively demonstrated that Mn-S_1_N_3_/SAE exerted superior anti-proliferation effects over Mn-N_4_/SAE, leveraging its photothermal-enhanced catalytic activities.

**Fig. 7:**
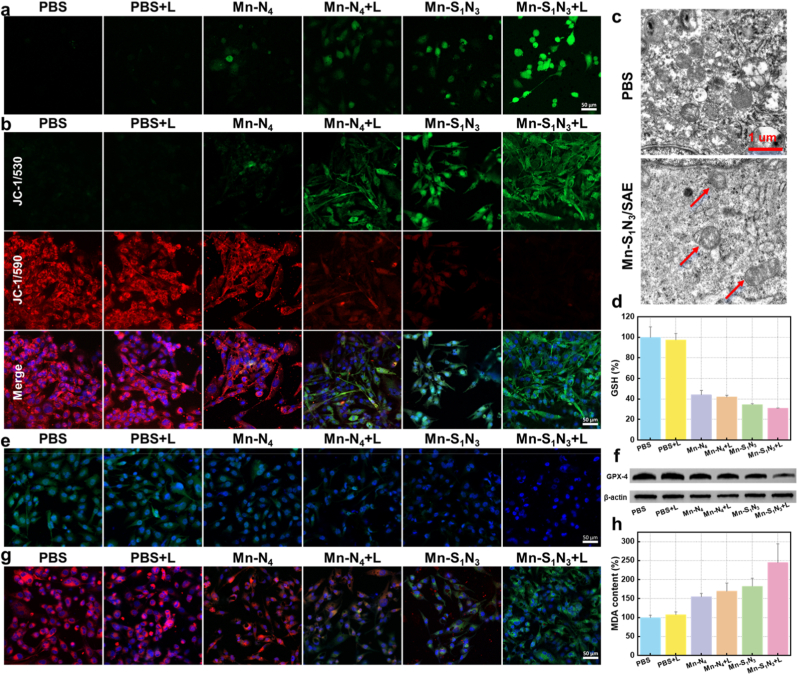
**Mechanism of Mn-S_1_N_3_/SAE and Mn-N_4_/SAE caused cells ferroptosis.** (a) DCF fluorescence images of U251 cells following varying treatments. (b) JC-1 fluorescence images of U251 cells following varying treatments. (c) Bio-TEM of U251 cells following varying treatments. (d) GSH levels in U251 cells following varying treatments. (e) The GPX4 expressions of U251 cells following varying treatments by immunofluorescence assay. (f) GPX4 expression in U251 cells incubated with varying formulations by WB. (g) The confocal images of fluorescent probe C11-BODIPY^581/589^-stained U251 cells following varying treatments. (h) MDA levels in U251 cells following varying treatments.

To elucidate the potential mechanism underlying the anti-proliferative effects of Mn-based SAEs, we explored their pathway on U251 cell. Considering the POD-mimicking activity of Mn-based SAEs and their photothermal properties, a fluorescent probe (2',7'-dichlorofluorescein diacetate, DCFH-DA) was employed to assess ROS levels in tumor cells. As shown in the Fig. 7a, CLSM imaging showed that Mn-S_1_N_3_/SAE exhibited significantly higher green fluorescence intensity than Mn-N_4_/SAE, demonstrating superior POD-like catalytic performance, which was further strengthened by laser irradiation. Elevated ROS levels in cells can trigger ferroptosis through the mitochondrial pathway, leading to disruption of the mitochondrial membrane potential (MMP). To assess changes in MMP, a mitochondria probe, 5,5’,6,6’-tetrachloro-1,1’,3,3’-tetraethyl-imida-carbocyanine iodide (JC-1) was employed, monitoring the fluorescence change from red to green. Confocal microscopy results displayed that both Mn-S_1_N_3_/SAE and Mn-N_4_/SAE triggered the loss of MMP (Fig. 7b). Importantly, the green fluorescence intensity boosted significantly upon laser irradiation, indicating severe mitochondrial damage, consistent with the observed ROS results. Further analysis using biological TEM (Bio-TEM) confirmed mitochondrial damage induced by Mn-S_1_N_3_/SAE in combination with laser treatment (Fig. 7c), a hallmark of ferroptosis. ROS oxidizes polyunsaturated fatty acids, leading to LPO. However, in tumor cells, high levels of reductive GSH rapidly counteract this process. Evaluation of the GSH consumption capacity revealed a significant reduction in GSH levels following treatment with Mn-based SAEs treatments (Fig. 7d). Importantly, Mn-S_1_N_3_/SAE exhibited a greater ability to deplete GSH compared to Mn-N_4_/SAE, with the effect further amplified upon laser irradiation. These results suggested that Mn-S_1_N_3_/SAE elevated oxidizing species by catalyzing H_2_O_2_ and reducing species by consuming GSH, thereby enhancing cellular oxidative stress and triggering LPO responses. We have learned that the consumption of GSH leads to the inactivation of GPX4, impairing lipid repair and subsequently inducing irreversible ferroptosis. In U251 cells, the expression of GPX4 protein was significantly diminished due to the GSH-consuming capacity of Mn-based SAEs (Fig. 7e and 7f and S32). As anticipated, both immunofluorescence and western blot (WB) assays revealed that Mn-S_1_N_3_/SAE reduced GPX4 expression more significantly than Mn-N_4_/SAE treatment, highlighting the enhanced catalytic activity resulting from S doping. The catalytic generation of •OH and subsequent GPX4 inactivation contribute to irreversible LPO, serving as a key marker for ferroptosis. To monitor LPO in cell membranes, a fluorescent kit, C11-BODIPY^581/589^, was employed (Fig. 7g and S33). Mn-S_1_N_3_/SAE resulted in a higher amplification in green fluorescence intensity, accompanied by a diminish in red fluorescence, proving that induction of LPO accumulation, which was further boosted by laser irradiation. To quantify LPO levels, malondialdehyde (MDA) levels were measured. Consistent with LPO results, Fig. 7h illustrated that Mn-S_1_N_3_/SAE induced the highest level of MDA. These results indicated that Mn-S_1_N_3_/SAE effectively promoted ferroptosis in U251 cells by inducing LPO accumulation and inactivating GPX4.

**Fig. 8:**
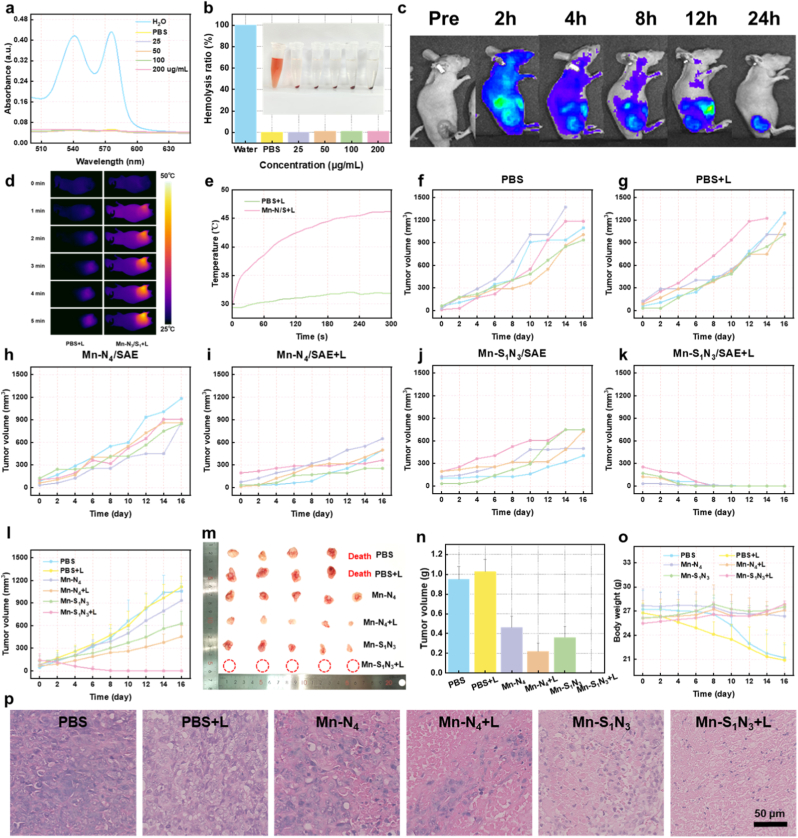
**Antitumor efficacy of Mn-S_1_N_3_/SAE and Mn-N_4_/SAE.** (a) Hemolysis analysis and (b) the corresponding quantification of red blood cells after treatment with various concentrations of Mn-S_1_N_3_/SAE. (c) The fluorescence images quantification of U251 tumor-bearing mice at various time points following the injection of Cy5.5-labeled Mn-S_1_N_3_/SAE. (d) Thermal imaging maps and (e) photothermal curves of the tumor site after exposure to 808 nm laser irradiation for 5 min. (f–l) Efficacy of subcutaneous tumor treatment in mice following various formulations. (m) The images and (n) tumor weights were collected from mice at the conclusion of the treatment. (o) Alterations in body weight of mice under diverse treatment conditions. (p) H&E staining of tumors subjected to various treatments.

Before evaluating the in vivo therapeutic efficacy of Mn-S_1_N_3_/SAE, its biocompatibility was assessed. Hemolysis assays demonstrated negligible hemolytic activity at concentrations up to 200 μg/mL (Fig. 8a and b). Furthermore, blood routine and blood biochemistry analysis revealed no significant signs of toxicity (Figs. S34 and 35). These results collectively indicate that Mn-S_1_N_3_/SAE possesses excellent hemocompatibility and robust biosafety. All animal experimental protocols were approved in advance by the Ethics Committee of Fujian Medical University. In addition, hematoxylin and eosin (H&E) staining was used to assess the normal cellular morphology of each tissue in vivo, revealing no obvious histological damage (Fig. S36). The IVIS imaging system was employed to determine Mn-S_1_N_3_/SAE accumulation in tumor sites. Cy5.5-labeled Mn-S_1_N_3_/SAE was intravenously administered, and fluorescence imaging results are shown in Fig. 8c and S37. The fluorescence signal in the tumor site continuously enhanced and maintained a high intensity for up to 24 h post-injection, indicating significant tumor accumulation of Mn-S_1_N_3_/SAE, likely due to the enhanced permeability and retention (EPR) effect (Fig. S38). Subsequently, taking advantage of the outstanding photothermal effect of Mn-S_1_N_3_/SAE, a laser irradiation (1 W/cm^2^) was used to the tumor site 24 h after intravenous injection, followed by thermal imaging. Thermal imaging displayed a quick increase in temperature at the tumor site compared to the control group, proving that Mn-S_1_N_3_/SAE showed a solid photothermal effect (Fig. 8d and e).

Considering the highly efficient photothermal-amplified catalytic activity of Mn-based SAEs, we developed a subcutaneous implantation model using U251 glioma to assess their therapeutic potential in vivo. When the average tumor volume reached approximately 80 mm^3^, the tumor-bearing mice were randomly divided into six groups (n = 5 per group): (I) control group, (II) laser group, (III) Mn-N_4_/SAE group, (IV) Mn-N_4_/SAE plus laser group, (V) Mn-S_1_N_3_/SAE group, and (VI) Mn-S_1_N_3_/SAE plus laser group. Body weight and tumor volume were monitored every two days. The relative tumor volume growth curves showed rapid tumor growth in the PBS and laser-only groups, suggesting that laser irradiation alone was ineffective (Fig. 8f–l). In contrast, mice treated with Mn-S_1_N_3_/SAE exhibited stronger tumor inhibition than those treated with Mn-S_1_N_3_/SAE, indicating the superior enzymatic activity of Mn-S_1_N_3_/SAE. Notably, the laser irradiation significantly enhanced the tumor inhibition effect, likely due to the photothermal-enhanced catalytic therapy, which eradicated tumor cells and improved the therapeutic efficiency of Mn-S_1_N_3_/SAE (Fig. 8m). Additionally, the mean tumor weight at the end of the treatment was significantly lower in the Mn-S_1_N_3_/SAE plus laser group compared to other groups, demonstrating the highest tumor suppression rate (Fig. 8n). The mean body weight of mice treated with Mn-based SAEs did not change significantly, indicating excellent in vivo biocompatibility (Fig. 8o). Kaplan-Meier survival analysis showed treatment with Mn-S_1_N_3_/SAE treatment increased survival rates, highlighting its substantial therapeutic effect on tumors (Fig. S39). Histological analysis using H&E staining and Terminal deoxynucleotidyl transferase dUTP nick end labeling (TUNEL) staining revealed that tumors collected in the Mn-S_1_N_3_/SAE plus laser group exhibited significant ferroptosis induction and inhibited cell proliferation (Fig. 8p and S40). Furthermore, immunofluorescence staining showed a marked reduction in GPX4 expression in the Mn-S_1_N_3_/SAE combined with laser irradiation group (Fig. S41). Together, these results revealed that Mn-S_1_N_3_/SAE combined with laser irradiation effectively induced tumor ferroptosis via the accumulation LPO and the inactivation of GPX4, suggesting its potential for use in tumor therapy.

## 3 Discussion

In conclusion, a S-doped Mn-S_1_N_3_/SAE was constructed with asymmetric Mn-S_1_N_3_ active sites anchored on C-N frameworks. The introduction of S atoms reshaped the local coordination environment, enhanced electron localization, and lowered the energy barrier, leading to amplified POD- and GSHOx-mimicking activities compared to the symmetric Mn-N_4_ configuration. DFT calculations revealed that the S-doping redistributed charge density around the Mn center, strengthened interactions with H_2_O_2_ and GSH, and promoted their activation. Moreover, S coordination slightly contracted the Mn-N bond length, which further facilitated charge transfer and catalytic efficiency. Both *in vitro* and in vivo results demonstrated that the Mn-S_1_N_3_/SAE significantly enhanced catalytic activity within the tumor microenvironment (TME), triggered tumor ferroptosis *via* LPO accumulation and GPX4 inactivation, which achieved greater tumor suppression than the Mn-N_4_/SAE analogue. Thus, these findings establish coordination asymmetry as an effective strategy to enhance the catalytic activity of SAEs and support their use in future catalytic therapy.

## 4 Methods

### 4.1 Synthesis of Mn-N_4_/SAE and Mn-S_1_N_3_/SAE

Mn@ZIF-8 precursors were synthesized using a host-guest strategy. 3.54 g 2-methylimidazole and 100 mg Mn(acac)_3_ were added into a 150 mL flask containing 60 mL methanol as solution A. 1.6 g Zn(NO_3_)_2_•6H_2_O was dissolved in 60 mL methanol as solution B. Then solution A was mixed with solution B, and the mixture was further stirred for 12 h. Then the Mn@ZIF-8 powders were collected by centrifugation, washed with methanol several times, and dried at 65 °C in a vacuum oven overnight.

And the Mn-S_1_N_3_/SAE was obtained by the two-step pyrolysis strategy. Firstly, the sublimed sulfur (S) powder (upstream) and Mn@ZIF-8 powder (downstream) were placed successively in a tubular furnace, and then pyrolysis at 400 °C for 1 h, followed by 950 °C for 3 h in an argon atmosphere. The Mn-N_4_/SAE was prepared by similar procedures except for adding S species.

• OH generation by Mn-N_4_/SAE and Mn-S_1_N_3_/SAE mediated catalytic reaction

Different concentrations of Mn-N_4_/SAE (or Mn-S_1_N_3_/SAE) and H_2_O_2_ (100 μM) were successively added into the PBS solutions with TMB (40 ug/mL), and the mixtures were shaken at 37 °C for 10 min. After centrifugation, the absorption spectra of supernatant were measured.

Mn-N_4_/SAE (or Mn-S_1_N_3_/SAE) and H_2_O_2_ (100 μM) were successively added into the TMB (40 ug/mL) solutions with different pH values, and the mixtures were shaken at 37 °C for 10 min. After centrifugation, the absorption spectra of supernatant were measured.

Electron spin resonance (ESR) analysis was carried out using DMPO as the spin trapper. To confirm the Mn-N_4_/SAE-mediated (or Mn-S_1_N_3_/SAE) •OH generation. 10 mM NaAc-HAc buffer solution (pH 4.3) containing 5 mM H_2_O_2_, Mn-S_1_N_3_/SAE (50 μg/mL), and 100 mM DMPO was ultrasonicated for 1 min. Then, the mixture was transferred to a quartz tube for ESR measurement.

### 4.2 GSH consume by Mn-N_4_/SAE and Mn-S_1_N_3_/SAE mediated catalytic reaction

Mn-N_4_/SAE (or Mn-S_1_N_3_/SAE) were mixed with GSH (10 mM) solutions at acidic pH, and the mixtures were shaken at 37 °C for 30 min. Finally, the DTNB (0.5 mg/ml) solution was added. After ultrafiltration, the GSH depletion was measured by the absorbance change at 415 nm.

### 4.3 Cellular uptake of Cy5.5-labeled Mn-S_1_N_3_/SAE

U251 cells were seeded in confocal dishes for 12 h. After 4 h of incubation with Cy5.5-labeled Mn-S_1_N_3_/SAE, the cells were co-stained with 20 μM Hoechst and 10 μM lysome-tracker for 20 min. The fluorescence imaging of tumor cells was imaged by CLSM.

### 4.4 Cytotoxicity assessments

U251 cells were planted for 24 h. Then, the cells were incubated with various concentrations of Mn-N_4_/SAE (or Mn-S_1_N_3_/SAE) without/with laser irradiation. After treatment for 24 h, the medium was replaced with fresh medium containing 10 μL CCK-8 and quantified by the absorbance at 450 nm using a microplate reader.

The U251 cells were seeded in confocal dishes and incubated for 12 h. After 24 h of exposure to Mn-N_4_/SAE (or Mn-S_1_N_3_/SAE) without/with laser irradiation (1 W/cm^2^, 808 nm), the cells were co-stained with calcein-AM and PI for 20 min. The fluorescence imaging of cells was observed directly by confocal microscopy.

U251 cells were seeded and incubated 12 h. Subsequently, the cells were exposed to Mn-N_4_/SAE (or Mn-S_1_N_3_/SAE) without/with laser irradiation. After co-staining with Annexin V-FITC and PI according to the manufacturer's protocols. The quantitative cell death was analyzed by flow cytometry.

### 4.5 In vitro reactive oxygen species (ROS) generation

U251 cells were seeded in confocal dish. After incubation for 12 h, the cells were treated with various formulation for 4 h. Then, the cells were co-stained with DCFH-DA (10 μM) and Hoechst (20 μM). The fluorescence imaging of cells was imaged by confocal microscopy.

### 4.6 Intracellular GSH

U251 cells were plated in 6-well plates and incubated for 24 h. Subsequently, the cells were exposed to Mn-S_1_N_3_/SAE without/with laser irradiation. The GSH contents were measured using a GSH kit. The assay was carried out according to the manufacturer's instructions.

### 4.7 Analysis of the change of MMP

To investigate the MMP, U251 cells were seeded and incubated for 24 h. Subsequently, the cells were exposed to Mn-N_4_/SAE (or Mn-S_1_N_3_/SAE) without/with laser irradiation. Then the cells were treated according to the JC-1 kit.

### 4.8 LPO initiated by Mn-N_4_/SAE and Mn-S_1_N_3_/SAE

U251 cells were seeded and incubated for 24 h. Subsequently, the cells were exposed to Mn-N_4_/SAE (or Mn-S_1_N_3_/SAE) without/with laser irradiation. Then the cells were stained with BODIPY^581/591^-C11 probe and Hoechst for 30 min.

### 4.9 Analysis of the change of MDA

To investigate the MDA, U251 cells were seeded and incubated for 24 h. Subsequently, the cells were exposed to Mn-N_4_/SAE (or Mn-S_1_N_3_/SAE) without/with laser irradiation. Then the cells were treated according to the MDA kit. The assay was carried out according to the manufacturer's instructions. The absorbance of 532 nm was measured by a microplate reader.

### 4.10 In vivo antitumor efficacy

Animal experiments were performed according to the protocol approved by The Ethical Committee of Fujian Medical University (IACUC FJMU2022-0608). The right hind legs of all mice were subcutaneously transplanted with U251 cells (1 × 10^6^ cells suspended in 100 μL of PBS). The tumor-bearing mice were used for antitumor treatment until the tumor volume reached about 70 mm^3^. Tumor volume = (tumor length) × (tumor width)^2^/2. U251 tumor-bearing nude mice were randomly divided into 6 groups (5 mice per group) and intravenously administrated with 10 mg/kg Mn-N_4_/SAE, Mn-S_1_N_3_/SAE or PBS. Living imaging was performed. After 24 h post-injection, the two laser exposure groups were irradiated (1 W/cm^2^, 808 nm) for 5 min. The surface temperature of the Mn-S_1_N_3_/SAE group and PBS group mice were recorded. The orbital blood of mousewere collected to exposed with Mn-N_4_/SAE, Mn-S_1_N_3_/SAE or PBS for hemolysis experiment. The tumor volumes and body weights were recorded every two days. After 16 days of treatment, one mice from each group were euthanatized for histological examination. Sacrificed mice were not included in the statistical results.

### 4.11 Statistical analysis

All quantitative data were expressed as the mean ± standard deviation (SD). Statistical analyses were performed using the Student's two-tailed *t*-test (∗P < 0.05, ∗∗P < 0.01, ∗∗∗P < 0.001, ∗∗∗∗P < 0.0001).

## CRediT authorship contribution statement

**Chenyu Ding:** Methodology. **Chengzhong Du:** Supervision. **Penghui Wei:** Data curation. **Yongrui Hu:** Formal analysis. **Hongjia Zheng:** Investigation. **Zhongyuan Shen:** Software. **Peisen Yao:** Data curation. **Lingjun Yan:** Resources. **Yang Zhu:** Writing – review & editing, Writing – original draft, Supervision.

## Declaration of competing interest

The authors declare no conflict of interest.

## Data Availability

Data will be made available on request.
