# Supplementary material for: Symmetry breaking unlocks superior reactivity in single atom catalytic therapy
Source: Mater Today Bio. 2025 Oct 27;35:102464. doi: 10.1016/j.mtbio.2025.102464 (PMC12596904; doi:10.1016/j.mtbio.2025.102464)
Supplement: Multimedia component 1 [file mmc1.docx]

Supporting Information

**Chemicals**

2,2’-azino-bis(3-ethylbenzothiazoline-6-sulfonic acid) diammonium salt (ABTS), Manganese cetylacetonate (Mn(acac)_3_), hydrogen peroxide (H_2_O_2_), acetic acid (HAc), sodium acetate (NaAc), and ethanol were purchased from Sinopharm Chemical Reagents (Shanghai, China). 3,3',5,5'-tetramethylbenzidine (TMB), and C11-BODIPY^581/591^ were provided by Sigma-Aldrich (St. Louis, USA). Hoechst 33342, 2′,7′-dichlorofluorescin diacetate (DCFH-DA), cell count kit-8 (CCK-8), annexin V-FITC/PI apoptosis detection kit, thiobarbituric acid (TBA), AM/PI, and 1,1',3,3'-tetraethyl-5,5',6,6'-tetrachloroimidacarbocyanine iodide (JC-1) were bought from Beyotime (Shanghai, China). Dulbecco's modified eagle medium (DMEM) was purchased from Hyclone (Logan, USA). 5,5-dimethyl-1-pyrroline N-oxide (DMPO) was bought from Dojindo (Dojindo). Cyanine 5.5 monosuccinimidyl ester (Cy5.5-NHS), ELISA, and annexin V-FITC/PI apoptosis detection kit were purchased from Beijing Solarbio Science & Technology Co., Ltd. (Beijing, China). Live & Dead Bacterial Staining Kit (Cat#40274ES60) and GMyc-PCR Mycoplasma Test Kit (Cat#40601) was purchased from Yeasen Biotechnology (Shanghai) Co., Ltd.. 20 mm glass-bottom dishes, and centrifuge tubes were obtained from NEST Biotechnology Co. Ltd. (Wuxi, China). Deionized (DI) water was obtained from a Milli-Q water purification system.

**Instruments**

Powder X-ray diffraction (XRD) patterns were recorded on a Rigaku Miniflex-600 diffractometer. Transmission electron microscope (TEM) images were taken by Hitachi-7700. High-angle annular dark field scanning transmission electron microscopy (HAADF-STEM) images were recorded by JEM-ARM200F (JEOL) TEM/STEM with a spherical aberration corrector. The energy-dispersive X-ray spectroscopy (EDS) mapping was performed by JEM-2100F. X-ray photoelectron spectroscopy (XPS) spectra were collected on scanning X-ray microprobe (PHI 5000 Verasa, ULAC-PHI). Scanning electron microscopy (SEM) images were taken by Nova NanoSEM 230. Fluorescence imaging was performed by confocal microscopy (Nikon C2). The absorption spectra were measured by a ultraviolet-visible (UV-vis) UH4150 spectrophotometer (Hitachi). Metal content was measured by using inductively coupled plasma mass spectrometer (ICP-MS, PlasmaQuad 3, Thermo Elemental). Hydrodynamic diameters and zeta potentials were determined by a Zetasizer nano ZS instrument (Malvern). Cancer cell apoptosis was monitored by using a flow cytometer (CytoFLEX, Beckman).


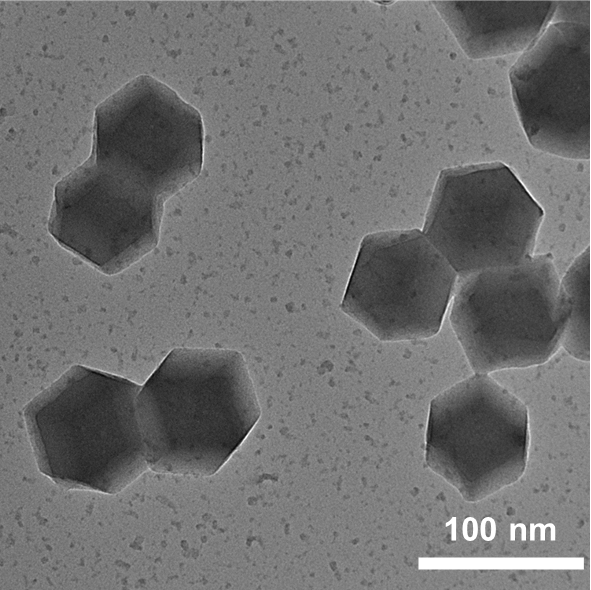


**Figure S1.** The TEM images of Mn@ZIF-8.

**
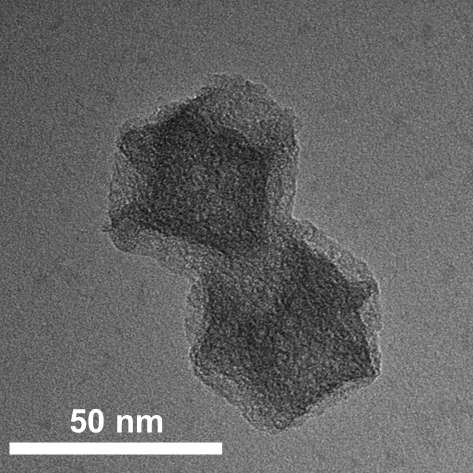
**

**Figure S2.** The TEM images of Mn-N_4_/SAE.

**
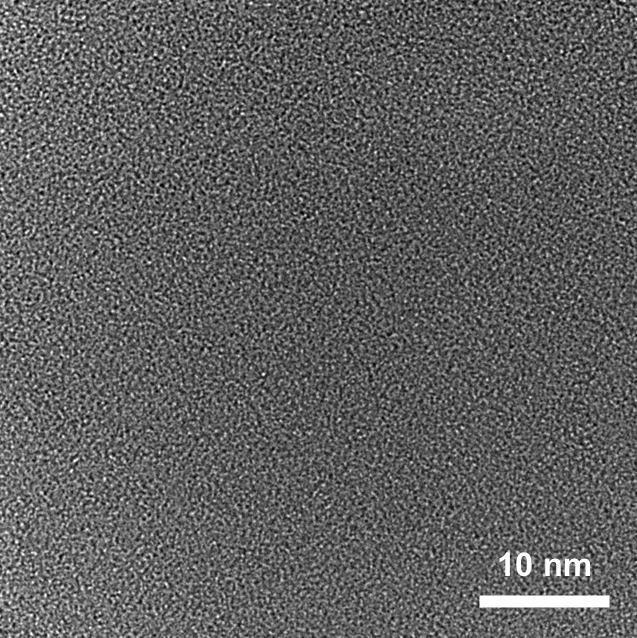
**

**Figure S3.** The HR-TEM of Mn-S1N3/SAE.

**
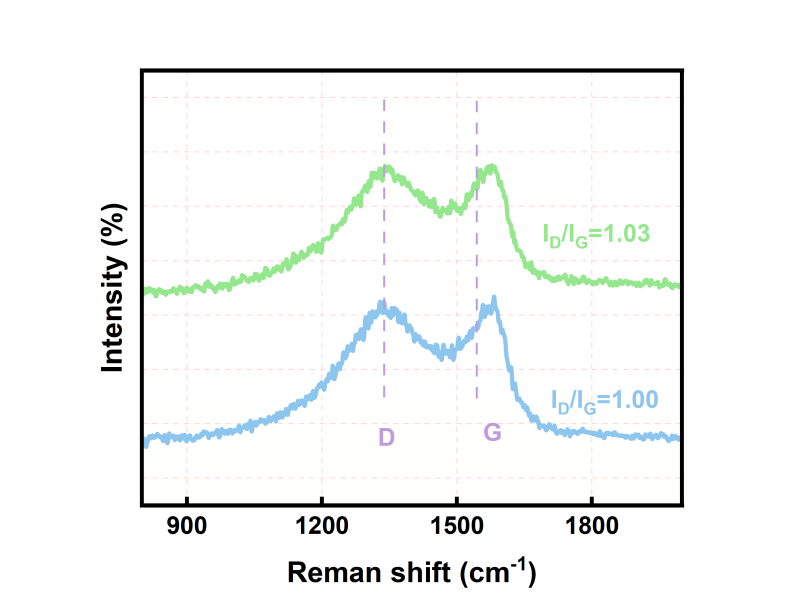
**

**Figure S4.** The Raman spectra of Mn-S_1_N_3_/SAE and Mn-N_4_/SAE.


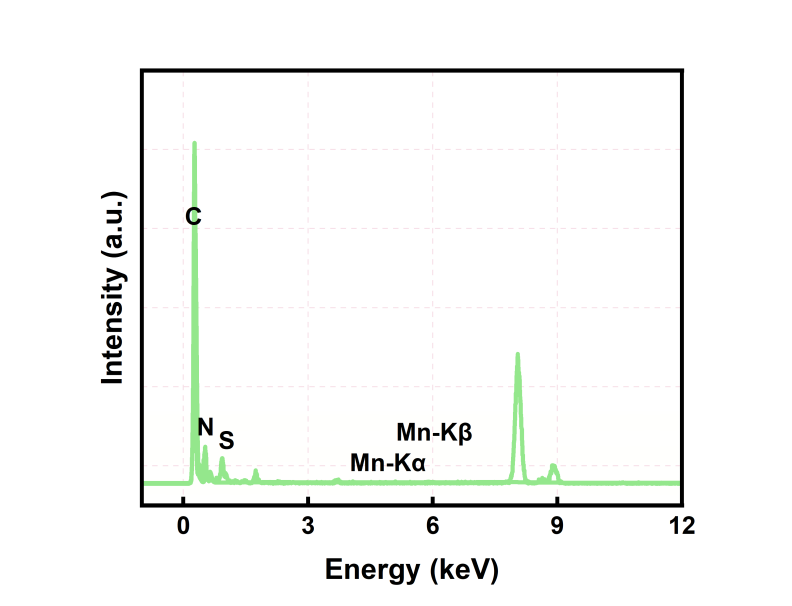


**Figure S5.** The EDX spectrum of Mn-S_1_N_3_/SAE.

**
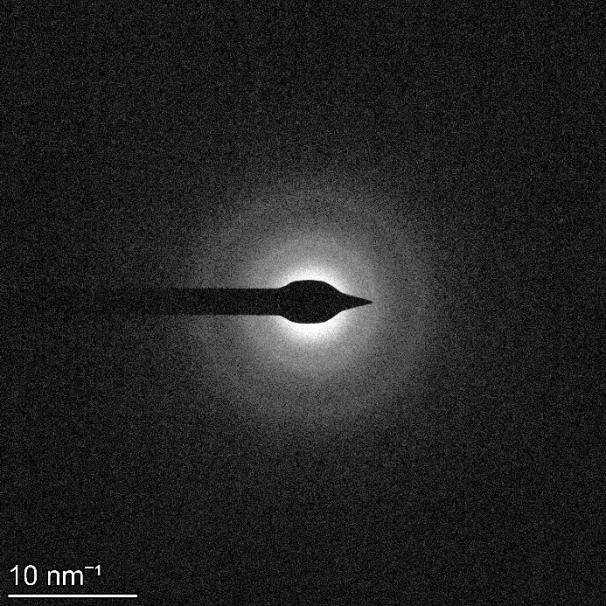
**

**Figure S6.** SAED pattern of the Mn-N_4_/SAE.


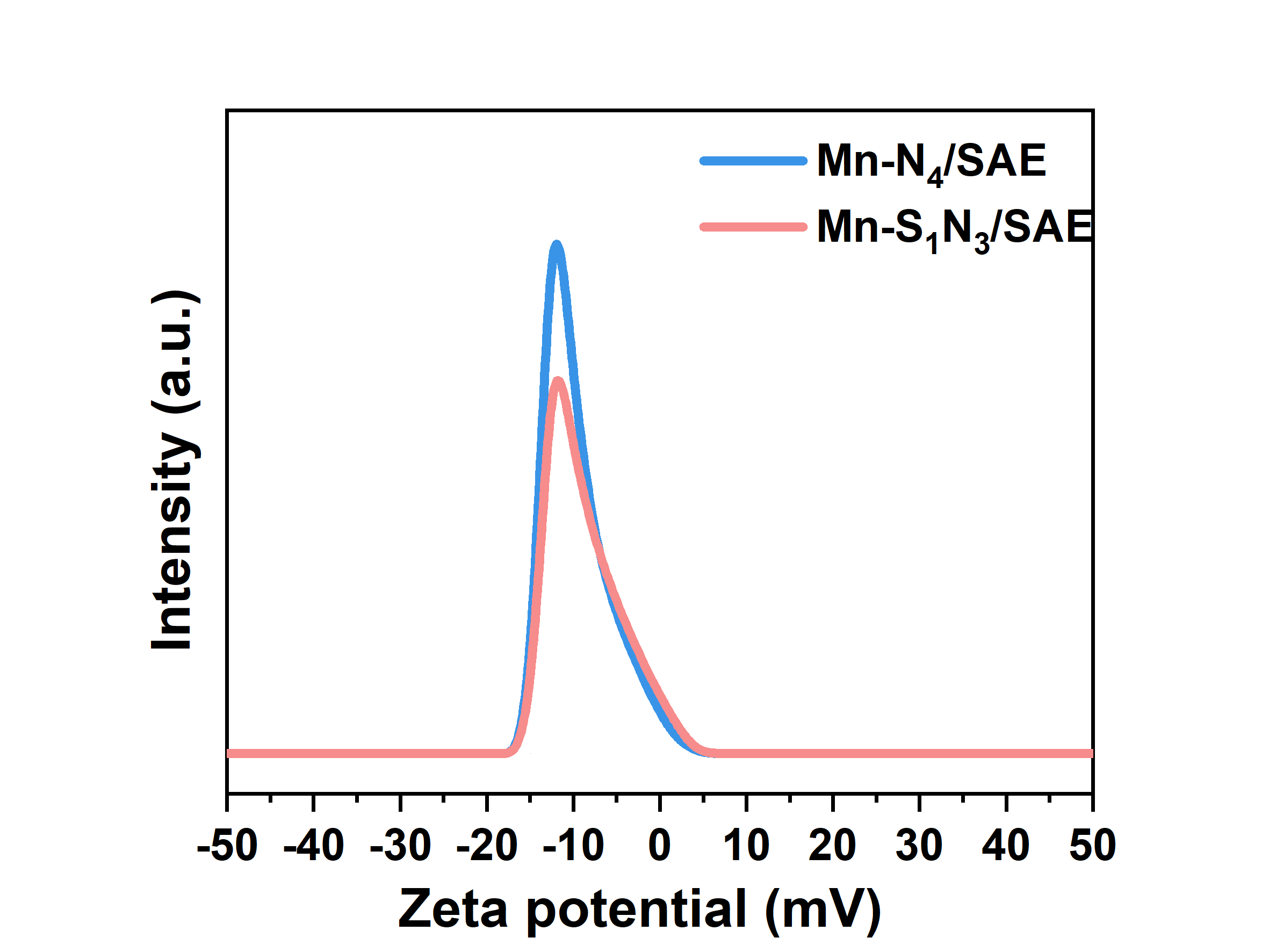


**Figure S7**. Zeta potential distribution of Mn-S_1_N_3_/SAE and Mn-N_4_/SAE.


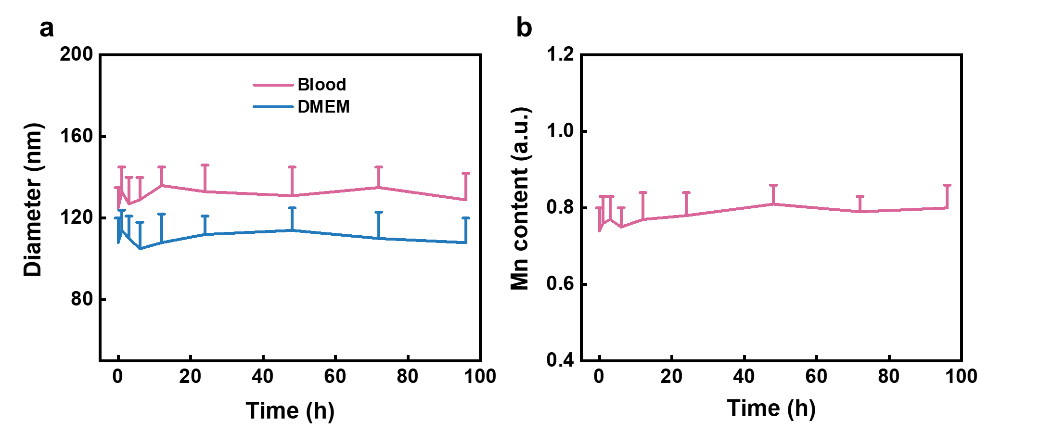


**Figure S8.** (a) The long-term stability of Mn-S_1_N_3_/SAE and (b) Mn atoms leakage.


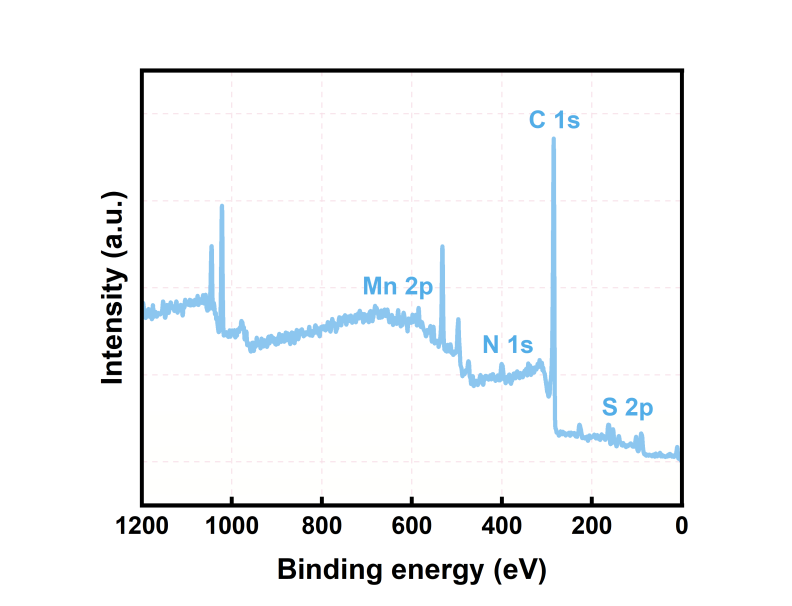


**Figure S9.** The survey XPS spectrum of Mn-S_1_N_3_/SAE.


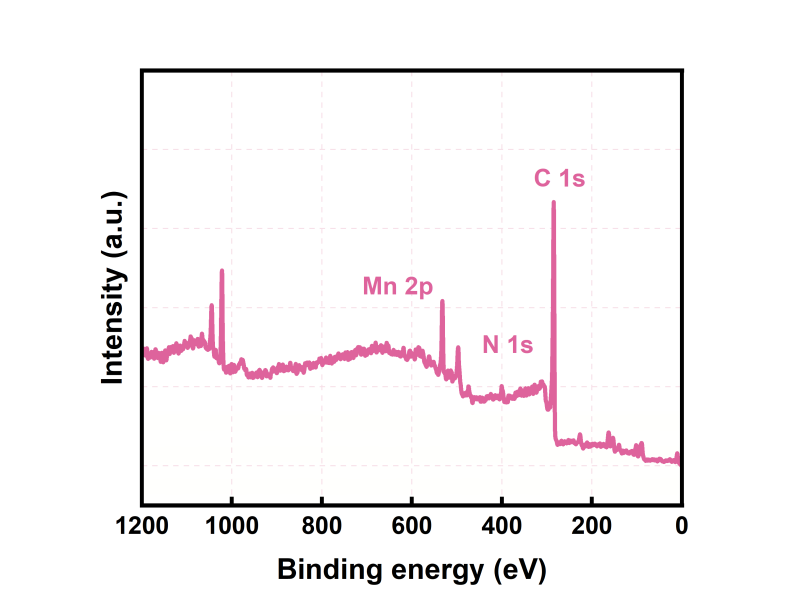


**Figure S10.** The XPS survey XPS spectrum of Mn-N_4_/SAE.


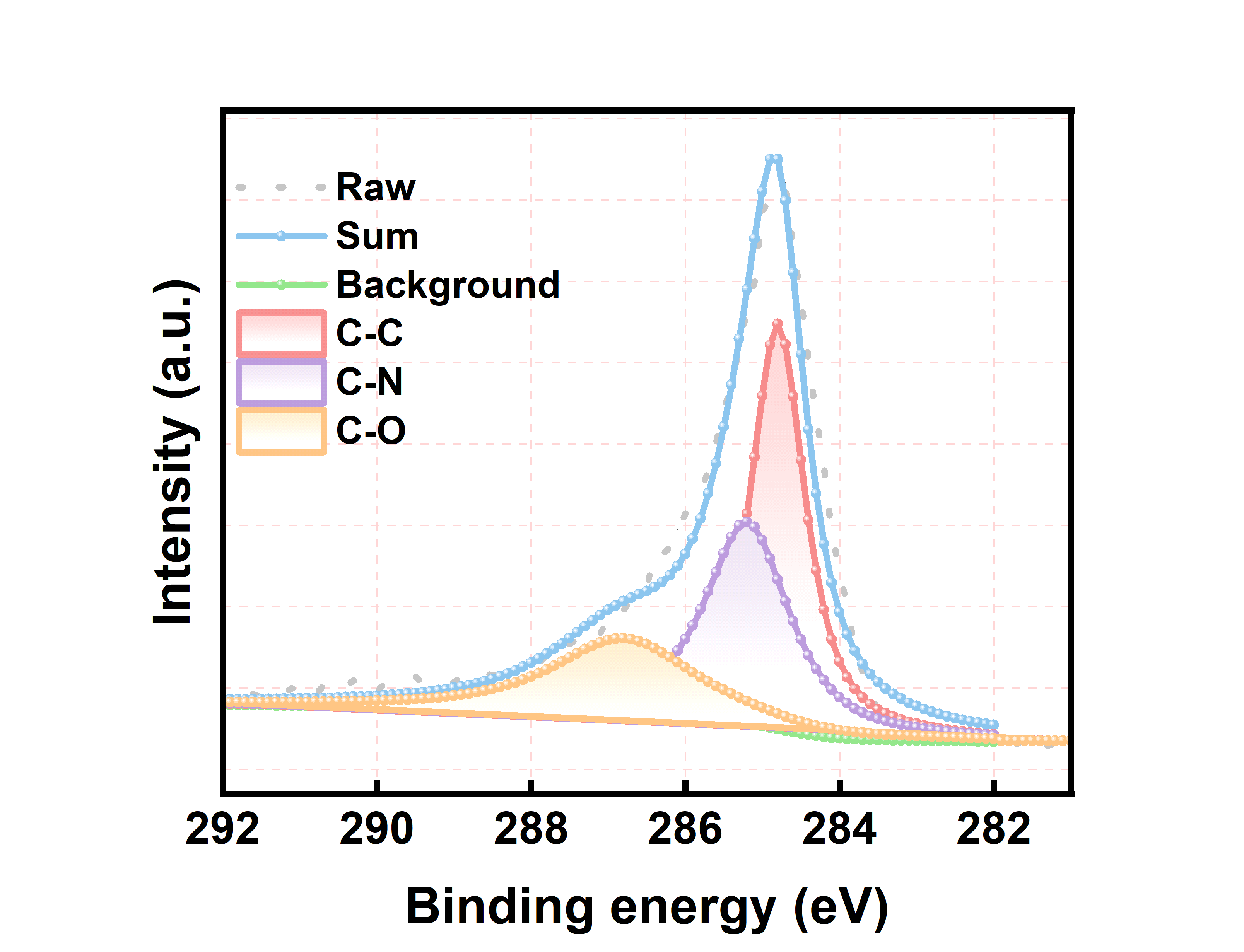


**Figure S11.** High-resolution C 1s XPS spectrum in Mn-N_4_/SAE.


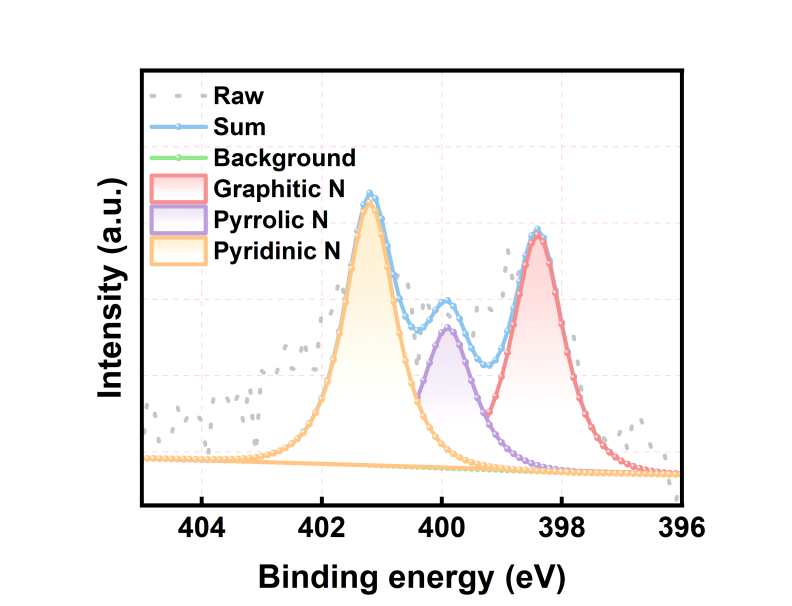


**Figure S12.** High-resolution N 1s XPS spectrum in Mn-N_4_/SAE.


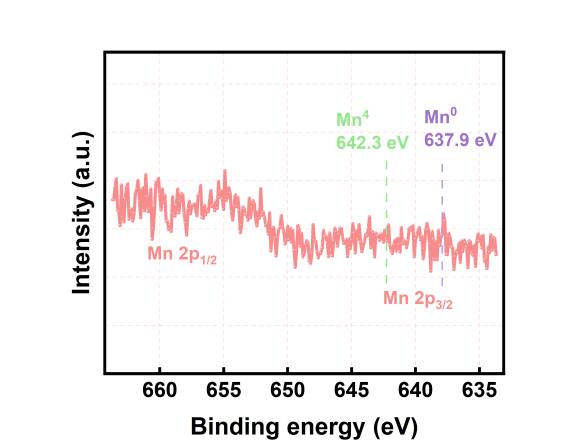


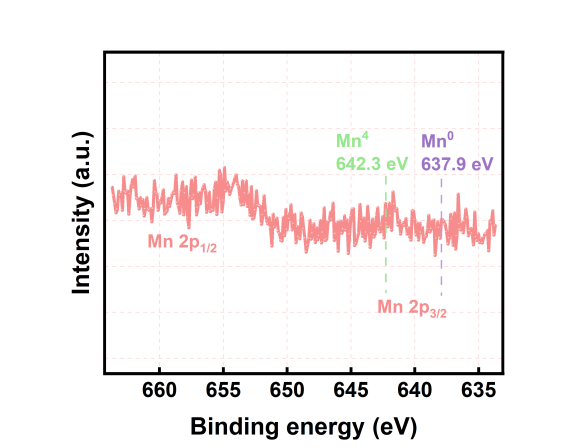


**Figure S13.** The Mn 2p XPS of Mn-S_1_N_3_/SAE and Mn-N_4_/SAE.


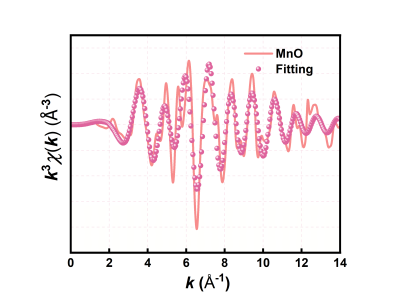


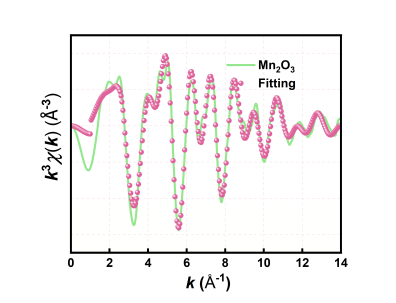


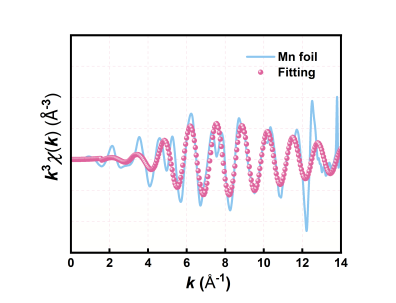


**Figure S14.** EXAFS fitting curves of MnO, Mn_2_O_3_, and Mn foil at the k space.

| **Sample** | **Path** | ***CNa*** | ***R*(Å)*b*** | ***σ*2 (Å2)*c*** | **Δ*E*0(eV)*d*** | ***R* factor** |
| --- | --- | --- | --- | --- | --- | --- |
| **Mn K-edge (*Ѕ*02=0.892)** | | | | | | |
| **Mn foil** | **Mn-Mn** | **12.0*** | **2.672±0.009** | **0.0045** | **9.4** | **0.0068** |
| **MnO** | **Mn-O** | **6.0±0.3** | **2.189±0.011** | **0.0083** | **-5.1** | **0.0172** |
|  | **Mn-Mn** | **12.0±0.3** | **3.135±0.007** | **0.0088** |  |  |
| **MnPc** | **Mn-N** | **4.0±0.3** | **1.964±0.007** | **0.0039** | **-2.7** | **0.0094** |
| **Mn-N_4_/SAE** | **Mn-N** | **3.9±0.3** | **2.067±0.018** | **0.0125** | **-7.8** | **0.0175** |
| **Mn-S_1_N_3_/SAE** | **Mn-N** | **3.1±0.3** | **2.068±0.018** | **0.0132** | **-7.9** | **0.0176** |
|  | **Mn-S** | **1.0±0.3** | **2.368±0.025** |  |  |  |

**Table S1.** EXAFS fitting parameters of various samples at the Mn K-edge (S0^2^ = 0.892).


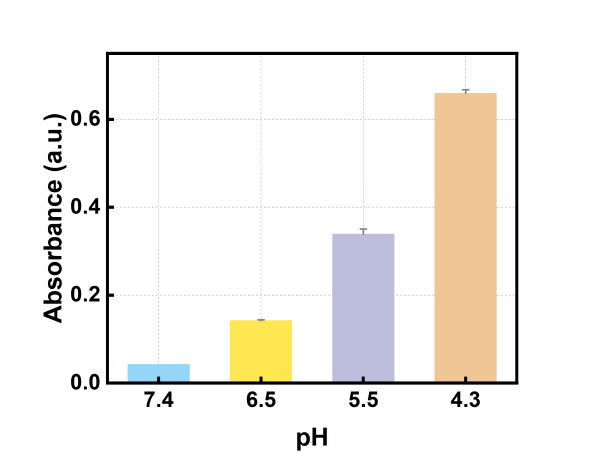

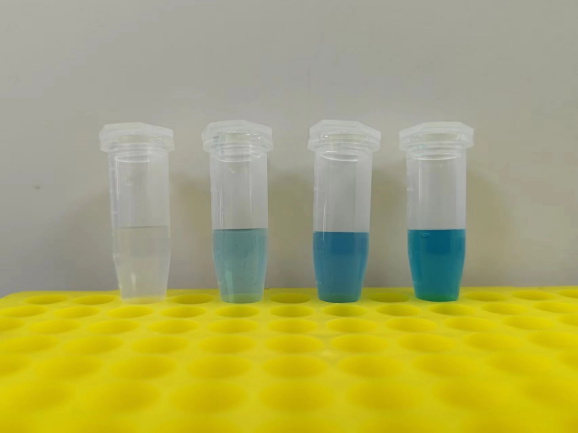


**Figure S15.** TMB assay for measuring POD-like activity of the Mn-S_1_N_3_/SAE at the different pH.


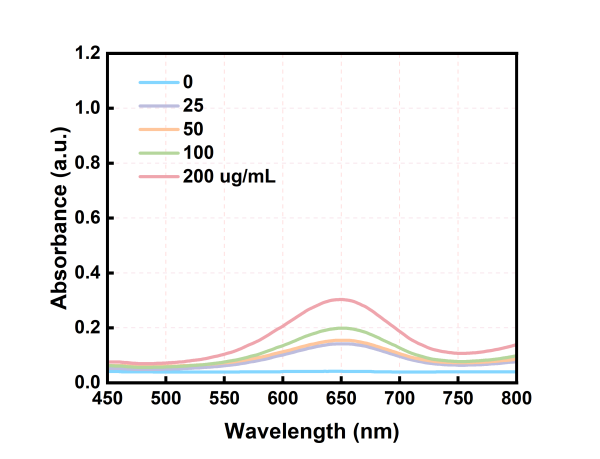

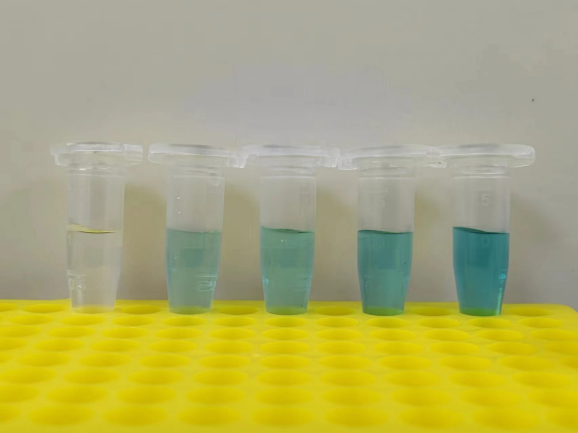


**Figure S16.** The absorbance of the produced oxide was measured by the Bio-Rad 650 microplate reader after incubating the different concentrations of Mn-N_4_/SAE with 40 ug /mL TMB for 5 min.


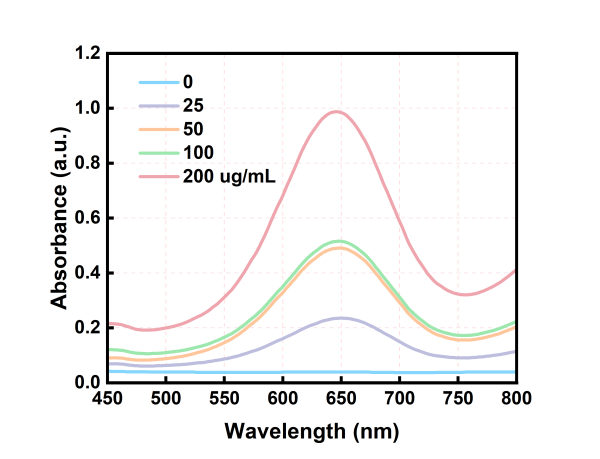

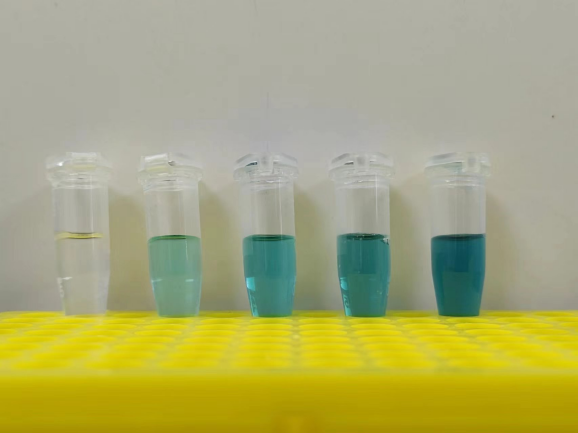


**Figure S17.** The absorbance of the produced oxide was measured by the Bio-Rad 680 microplate reader after incubating the different concentrations of Mn-S_1_N_3_/SAE with 40 ug /mL TMB for 5 min.


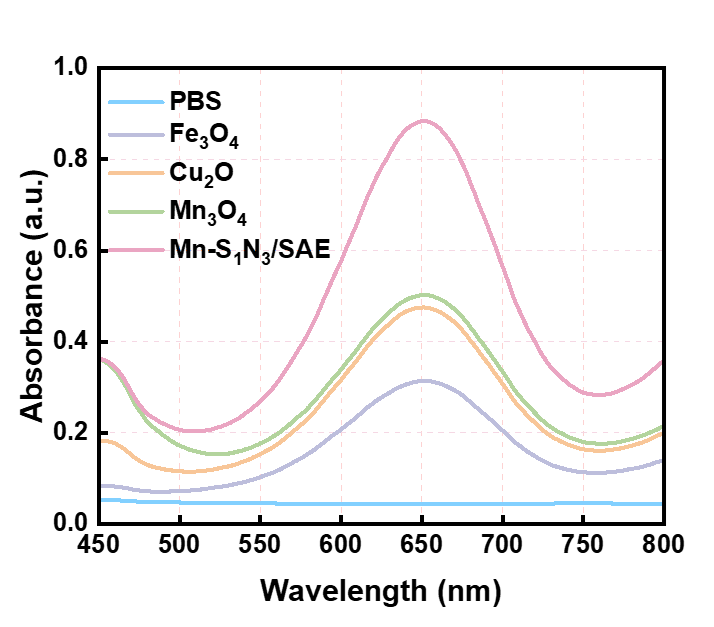


**Figure S18.** The POD-like activity of different nanozymes based on TMB assay.


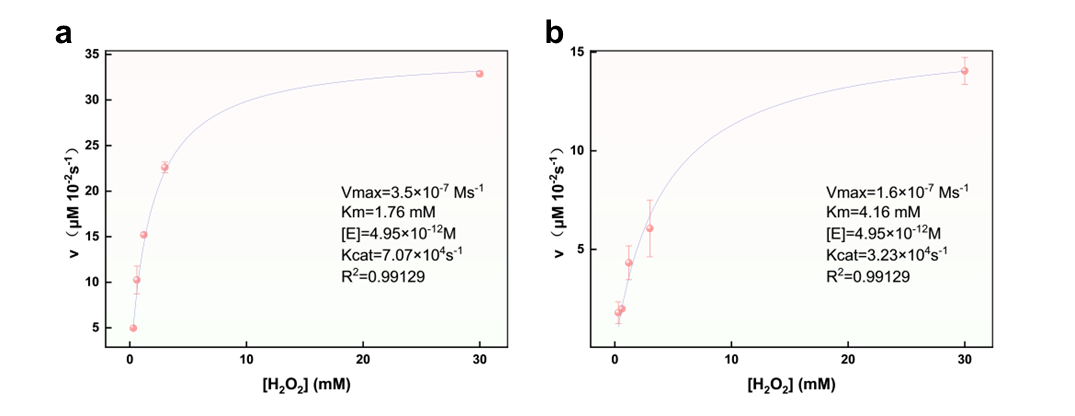


**Figure S19.** (a) The Km and Kcat of Mn-S_1_N_3_/SAE and (b) Mn-N_4_/SAE.


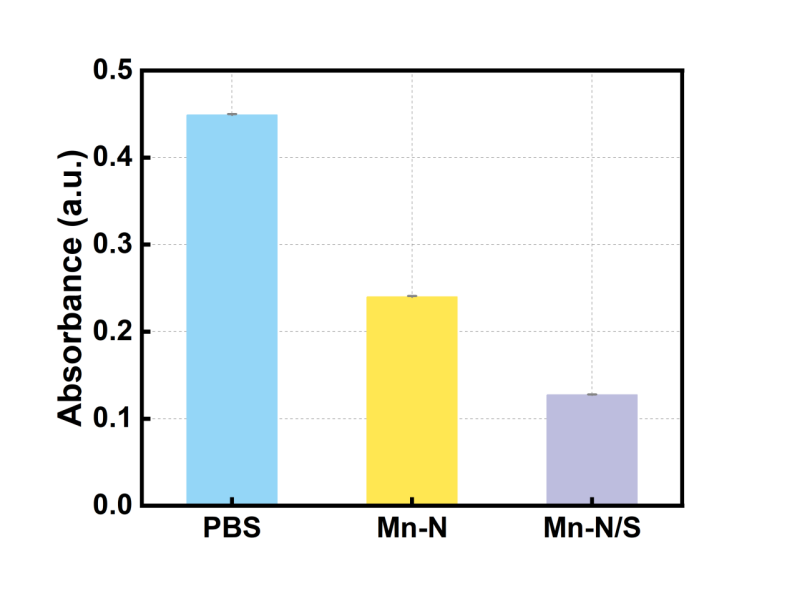


**Figure S20.** The absorbance of the produced oxide at 650 nm was measured by the Bio-Rad 650 microplate reader after incubating the different concentrations of Mn-S_1_N_3_/SAE and Mn-N_4_/SAE with MB for 2h.


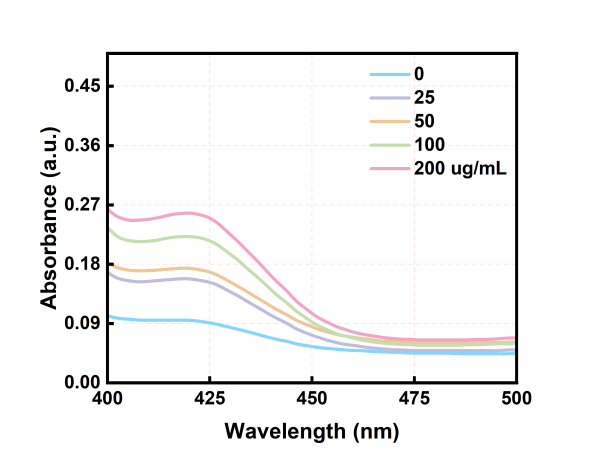

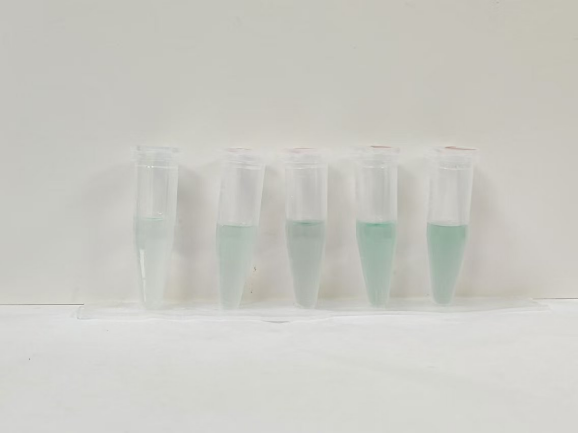


**Figure S21.** The absorbance of the produced oxide was measured by the Bio-Rad 680 microplate reader after incubating the different concentrations of Mn-N_4_/SAE with ABTS for 10 min.


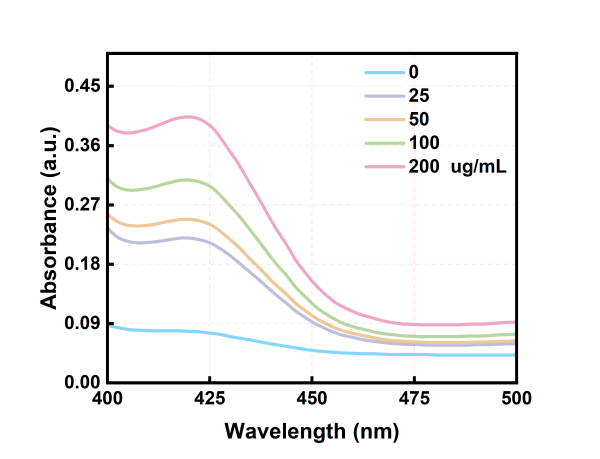

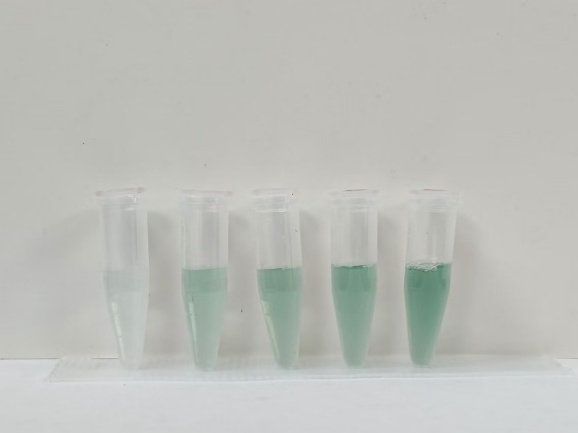


**Figure S22.** The absorbance of the produced oxide was measured by the Bio-Rad 680 microplate reader after incubating the different concentrations of Mn-S_1_N_3_/SAE with ABTS for 10 min.


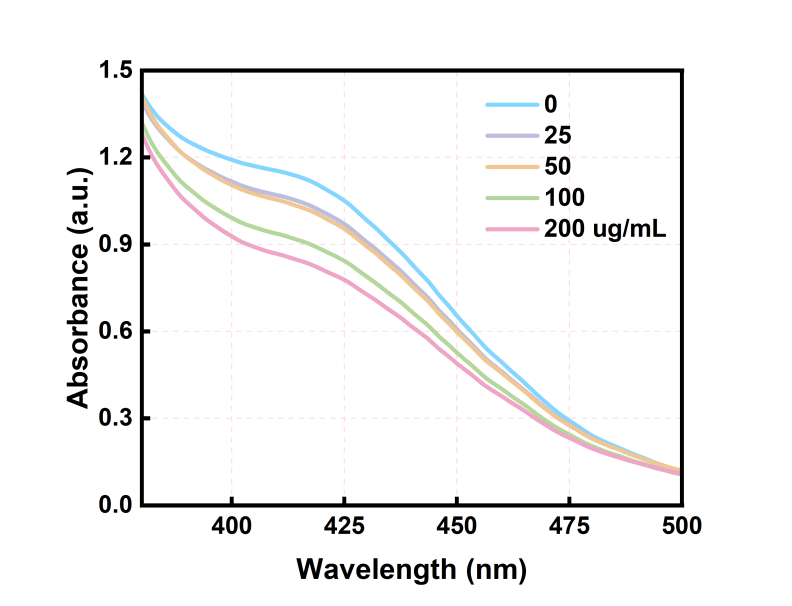


**Figure S23.** The absorbance of the produced oxide was measured by the Bio-Rad 680 microplate reader after DTNB incubated with Mn-N_4_/SAE in the presence of GSH.


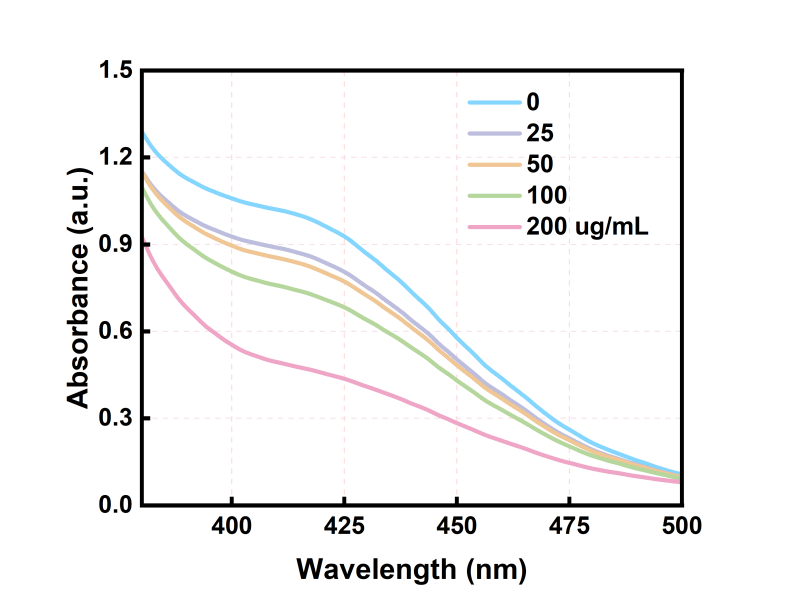


**Figure S24.** The absorbance of the produced oxide was measured by the Bio-Rad 680 microplate reader after DTNB incubated with Mn-S_1_N_3_/SAE in the presence of GSH.


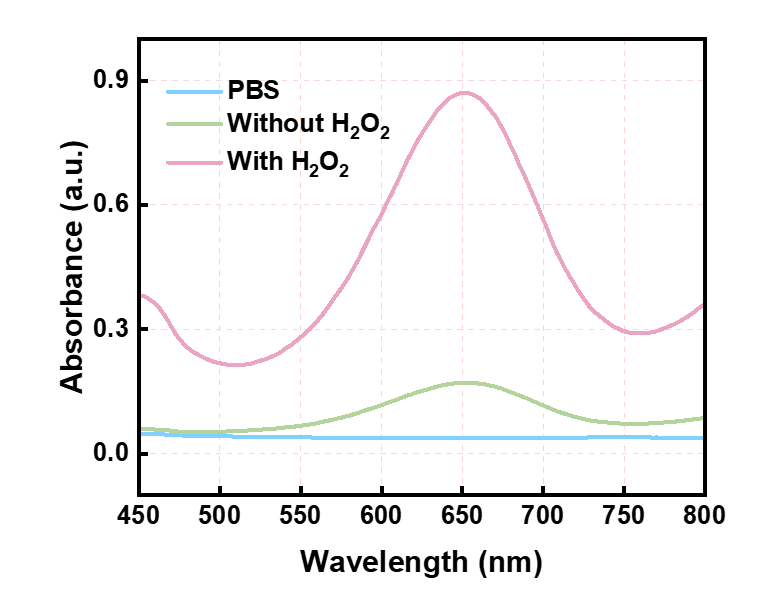


**Figure S25**. UV-vis spectrum of TMB incubated with different formulations.


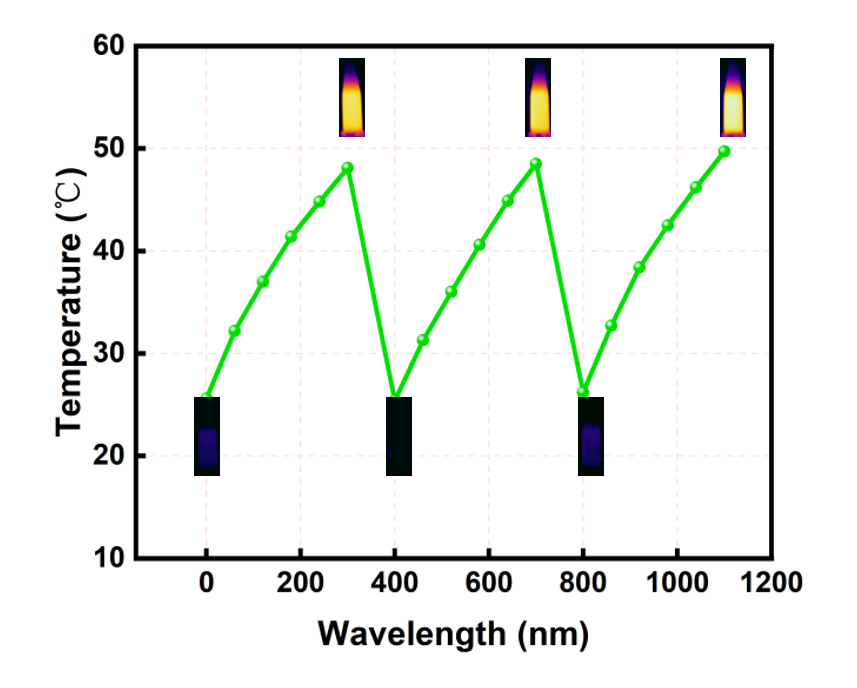


**Figure S26.** Repeated three cycles to investigate the photothermal stability of the material.


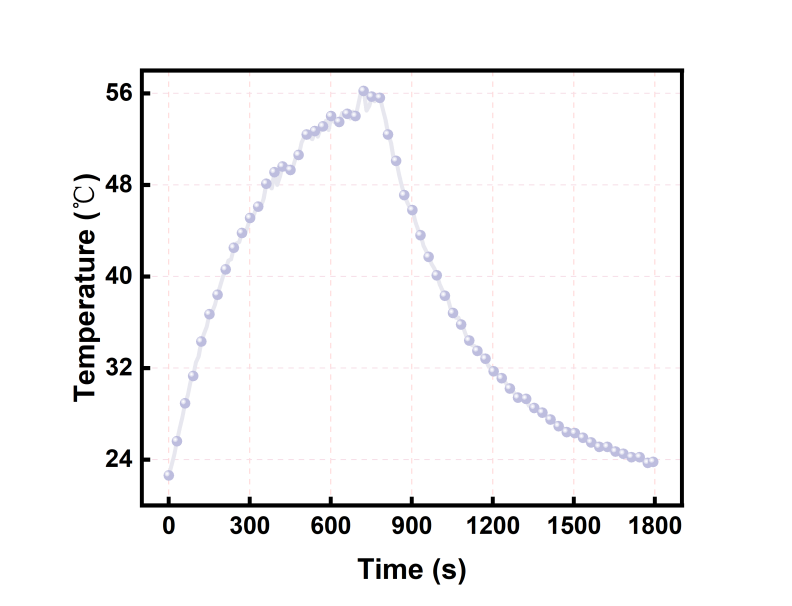


**Figure S27.** Photothermal conversion efficiency of Mn-S_1_N_4_/SAE.


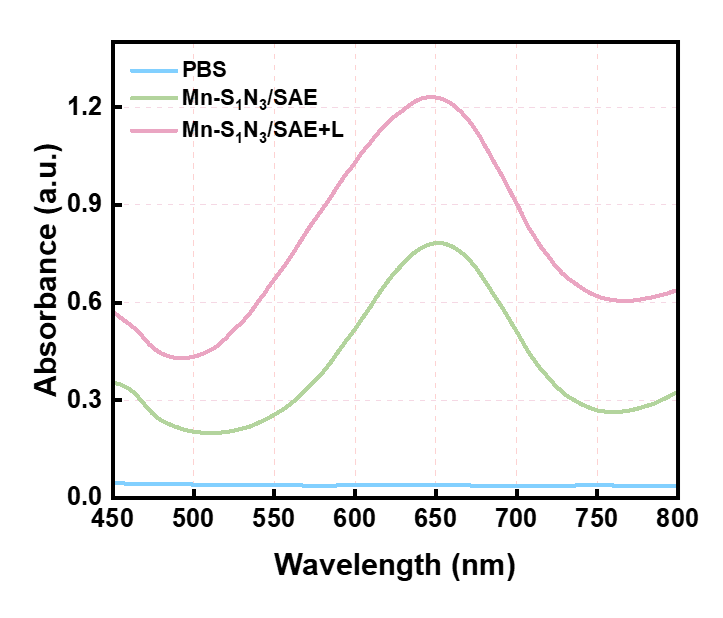


**Figure S28**. UV-vis spectrum of TMB plus H_2_O_2_ following different treatments.


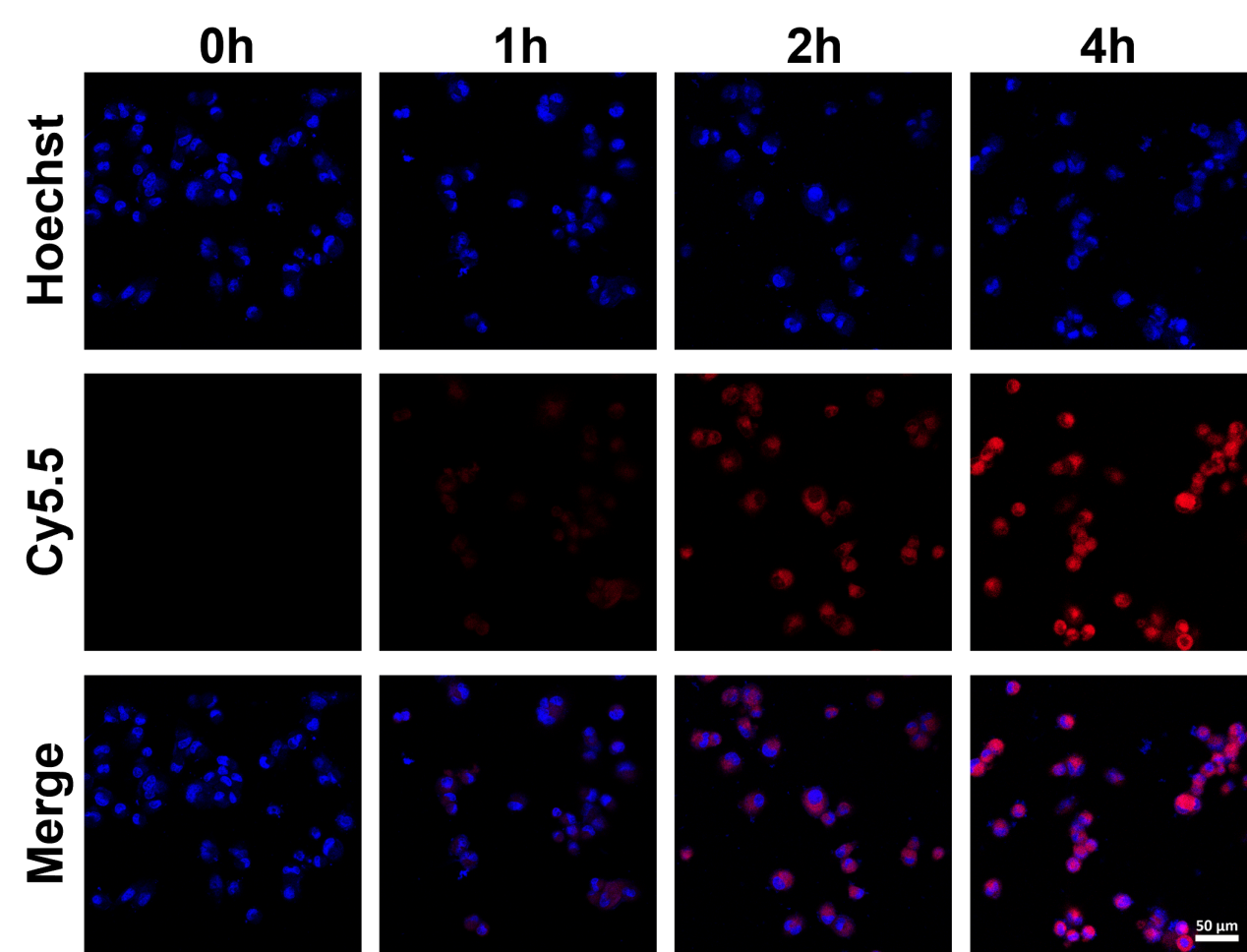


**Figure S29.** The CLSM images of tumor cells incubated with Cy5.5-labeled Mn-S_1_N_3_/SAE.


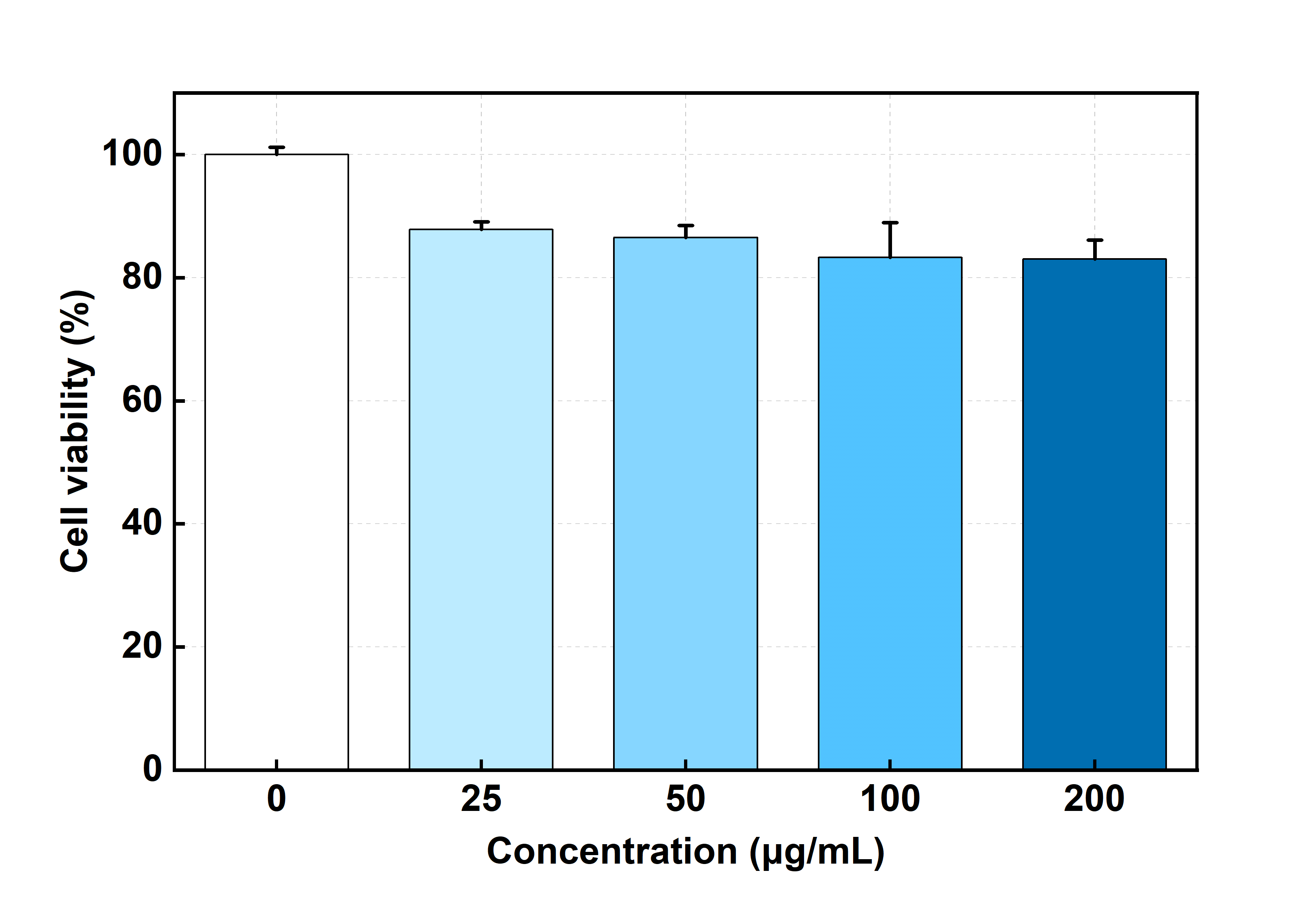


**Figure S30.** The cell viability of non-cancerous cells after 24 h treatment under different Mn-S_1_N_3_/SAE concentrations.


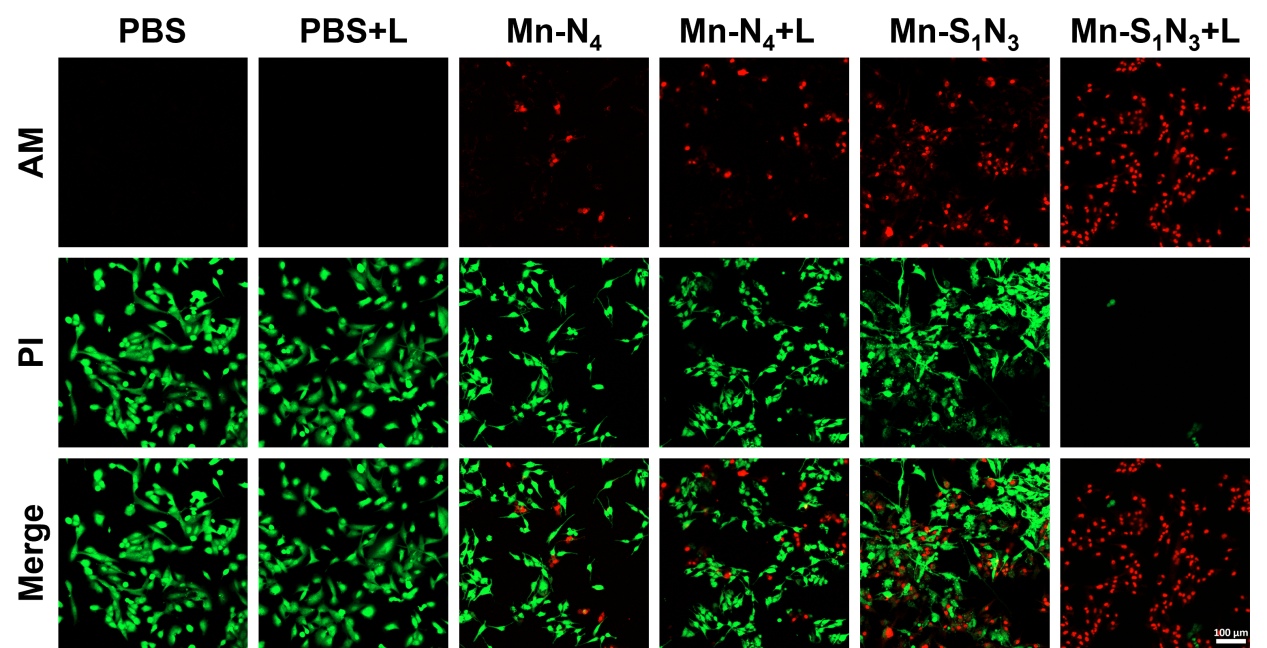


**Figure S31.** Calcein-AM/PI co-stained U251 cells incubated with various formulations.


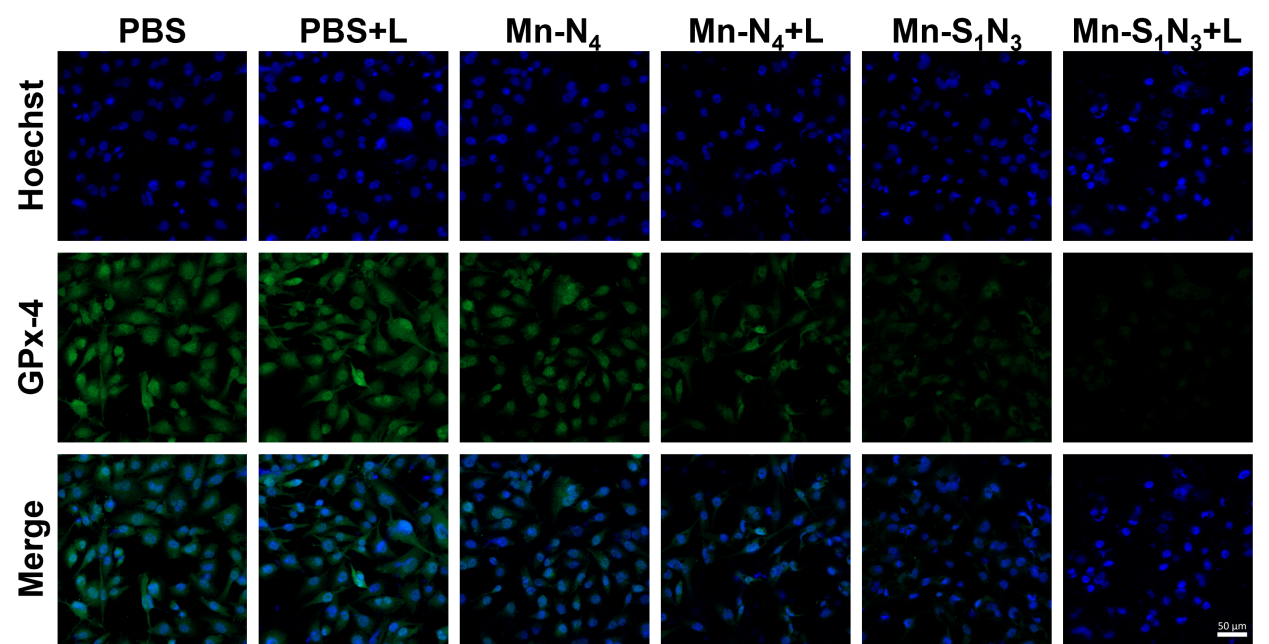


**Figure S32.** CLSM images of GPX4 expression in U251 cells treated with different formulations.


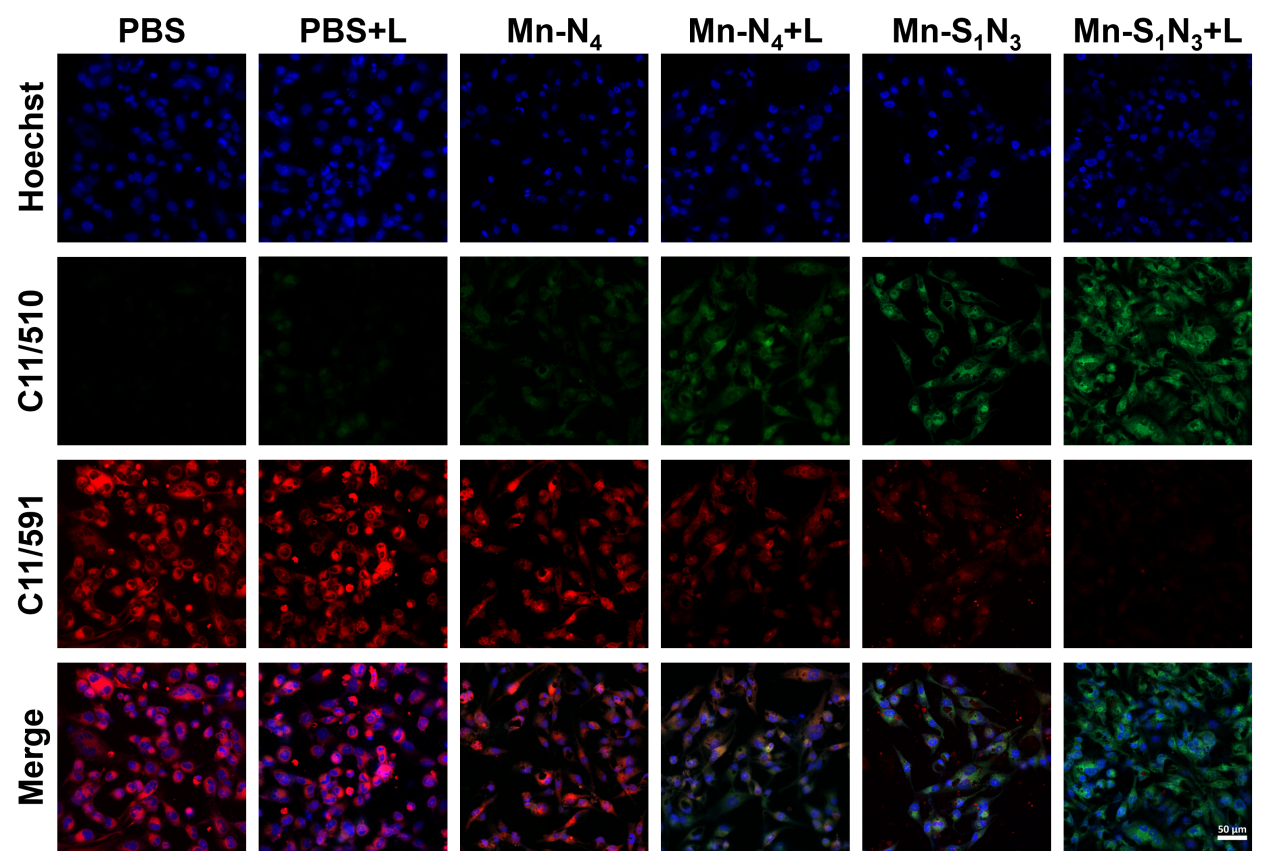


**Figure S33** The CLSM images of ratiometric fluorescent probe C11-BODIPY^581/589^-stained U251 cells after exposure to different formulations.


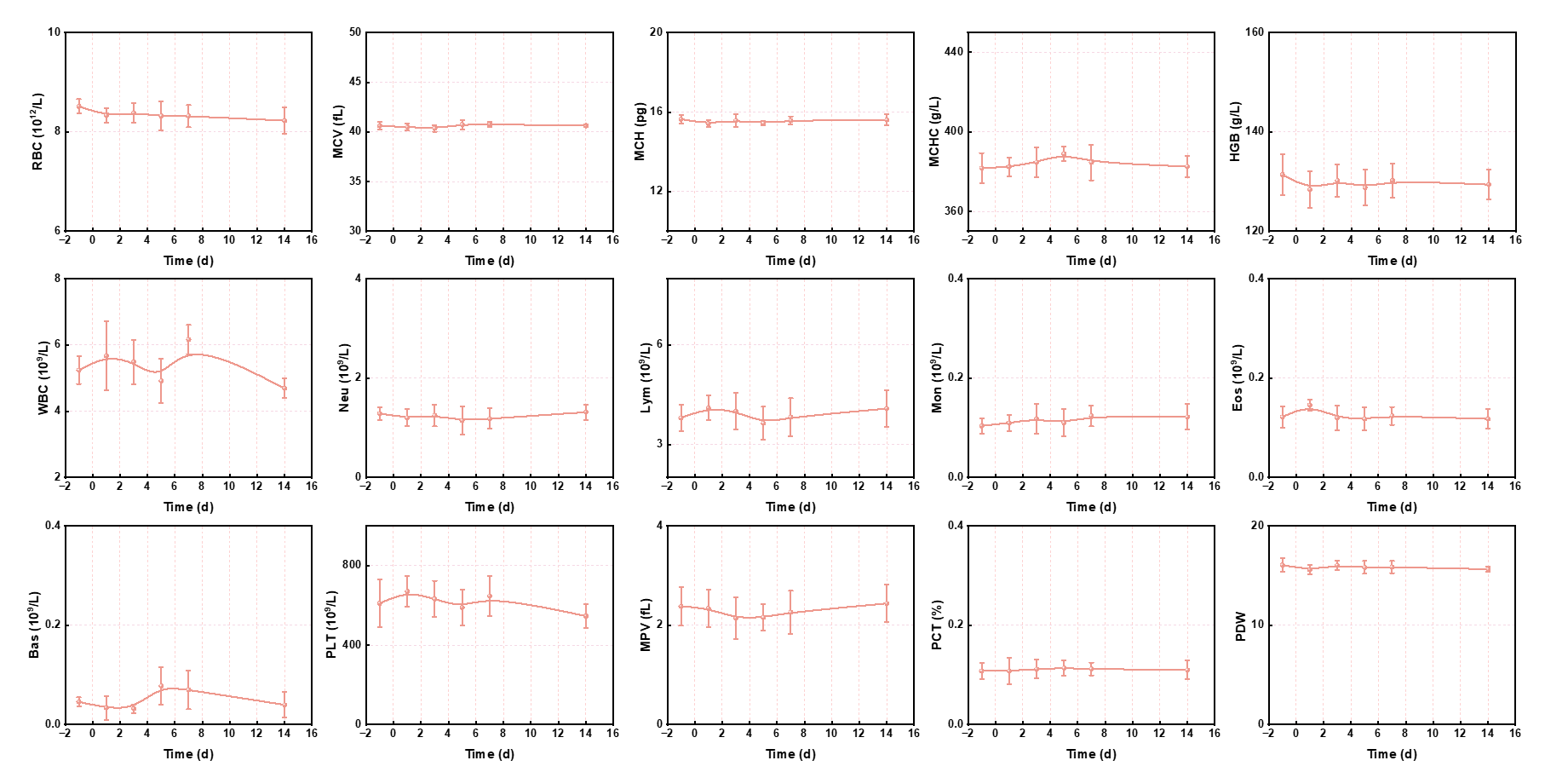


**Figure S34.** Trend graphs of different routine blood test indices over time after mice were intravenously injected with Mn-S_1_N_3_/SAE via caudal vein. (a) RBC, Red Blood Cells. (b) WBC, White Blood Cells. (c) HGB, Hemoglobin. (d) Neu, Neutrophils. (e) Mon, Monocytes. (f) MPV, Mean Platelet Volume. (g) MCHC, Mean Corpuscular Hemoglobin Concentration. (h) HCT, Hematocrit. (i) PLT, Platelets. (j) PCT, Plateletcrit. (k) Lym, Lymphocytes. (l) MCV, Mean Corpuscular Volume.


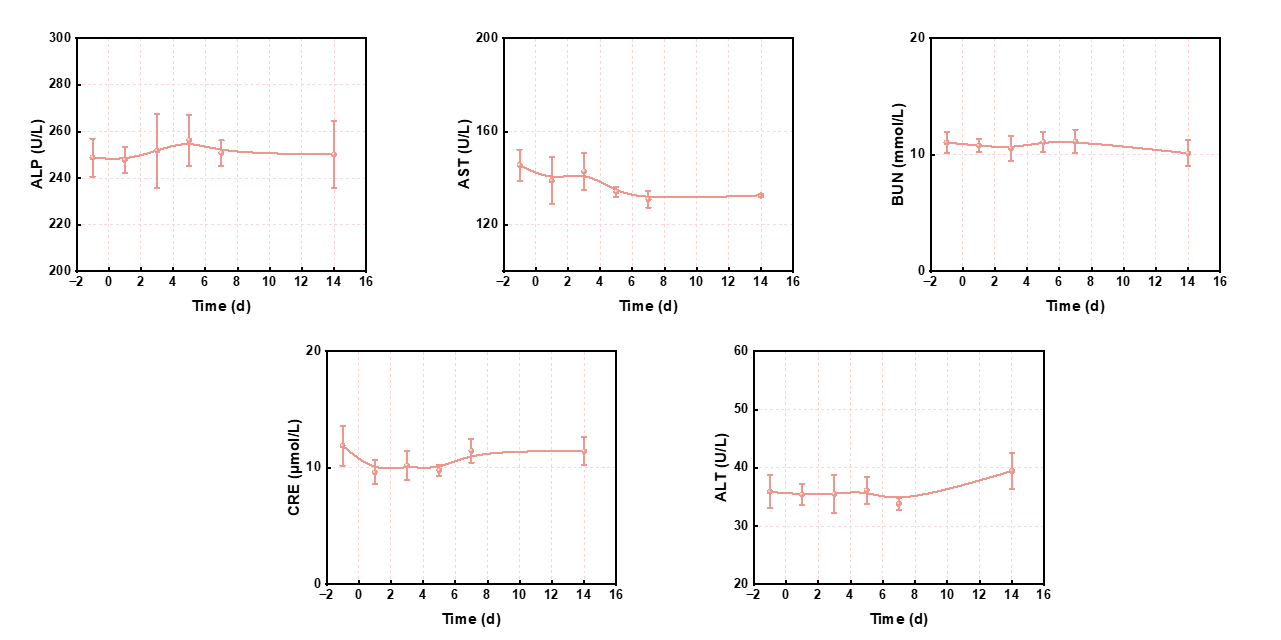


**Figure S35.** Trend graphs of different biochemical markers over time after mice were intravenously injected with Mn-S_1_N_3_/SAE via caudal vein.


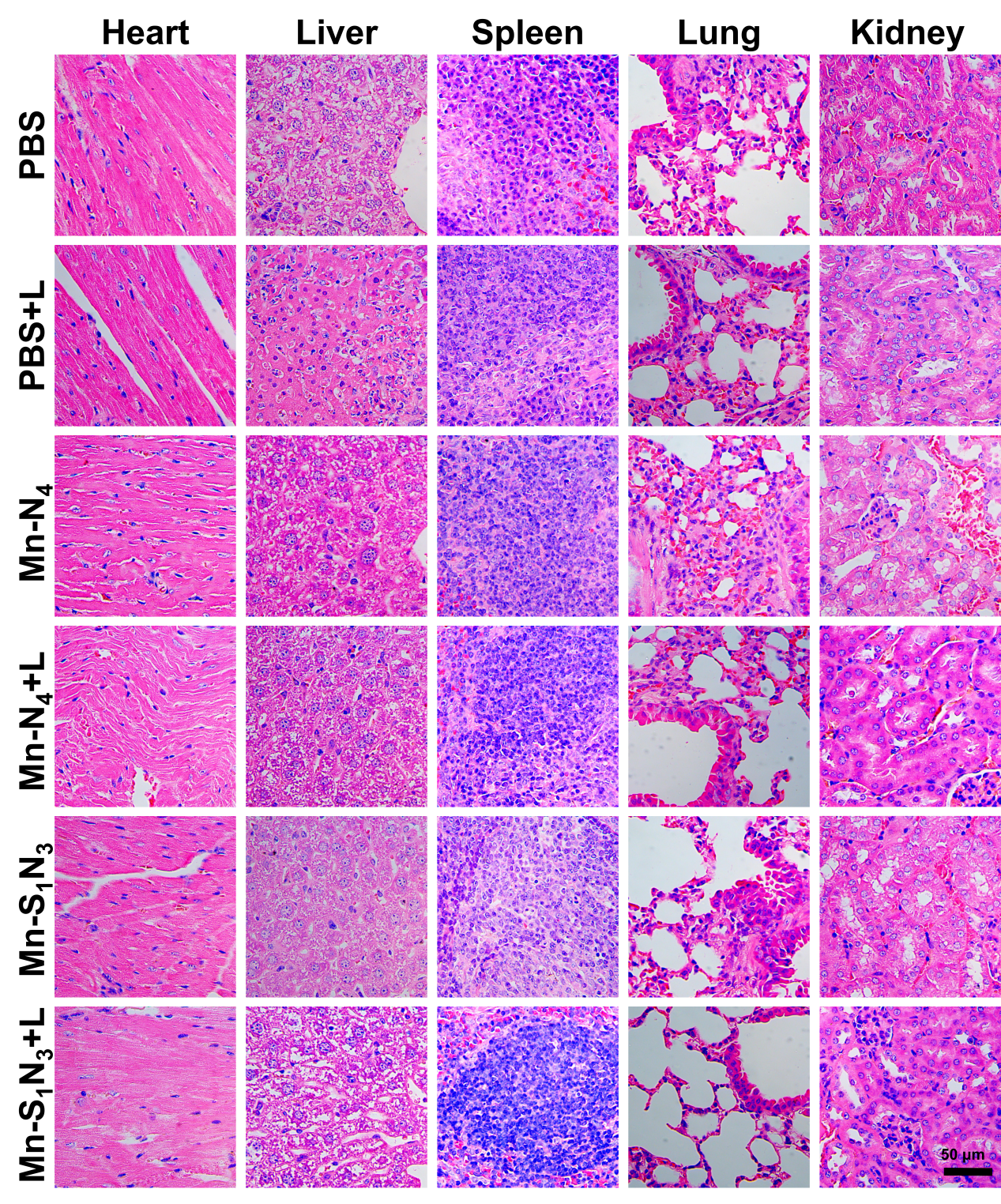


**Figure S36.** H&E staining of the major organs.


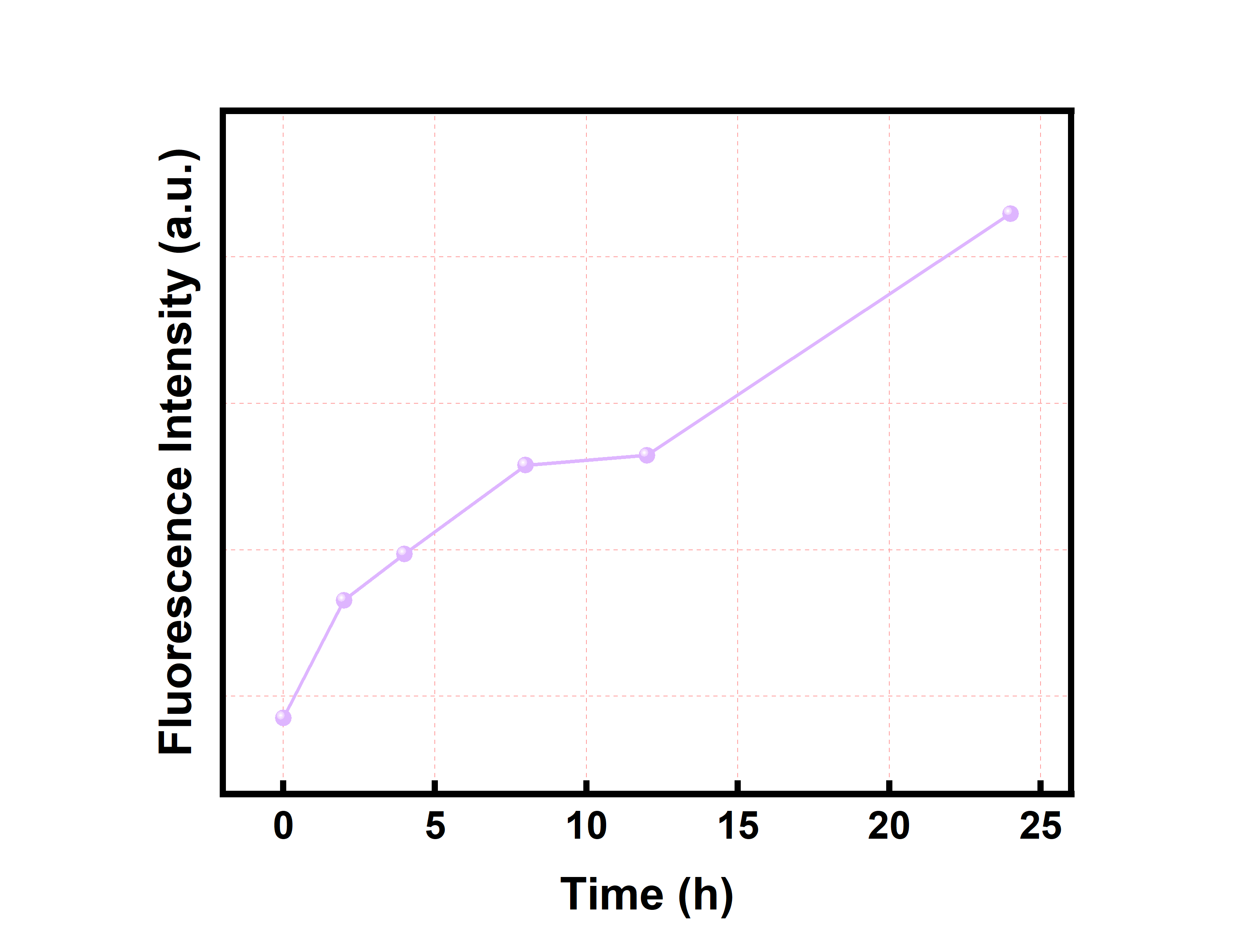


**Figure S37.** The fluorescence corresponding quantification of U251 tumor-bearing mice at various time points following the injection of Cy5.5-labeled Mn-S_1_N_3_/SAE.


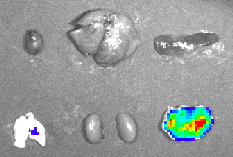


**Figure S38** Fluorescence images of the major organs (Tumor, heart, liver, spleen, lung, kidney) and tumor collected from the tumor bearing mice at 24 h after intravenous injection.


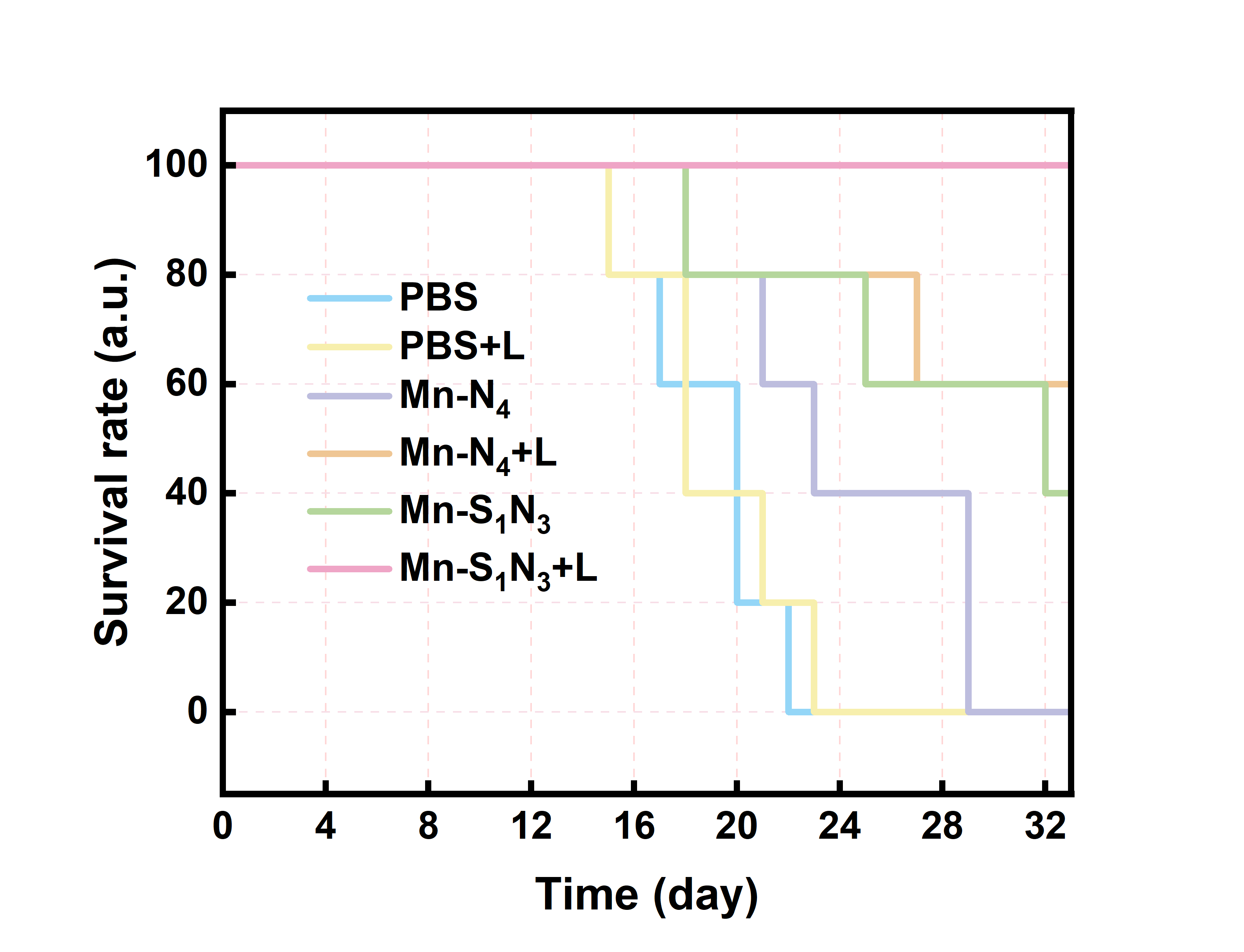


**Figure S39.** Kaplan-Meier survival analysis of tumor-bearing mice.


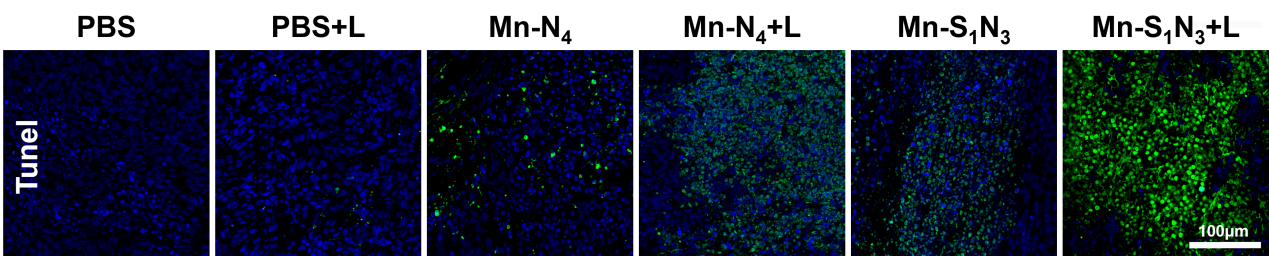


**Figure S40.** TUNEL staining of tumor tissue.


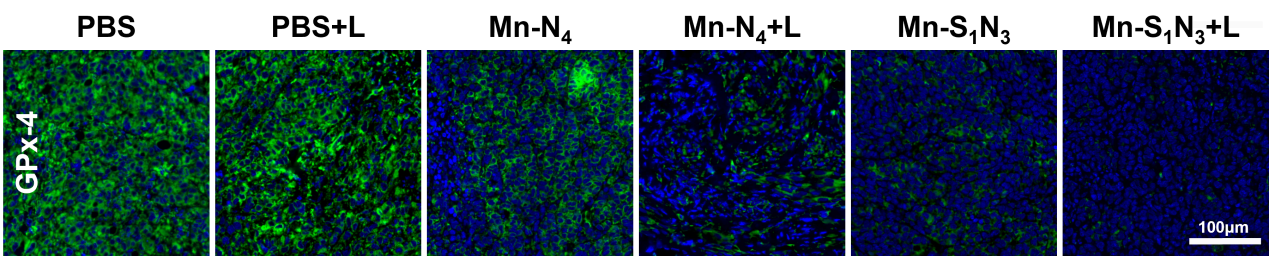


**Figure S41.** GPX4 immunofluorescence staining of tumor tissue.
